# Electrochemical detection of urinary microRNAs via sulfonamide-bound antisense hybridisation

**DOI:** 10.1016/j.snb.2017.06.069

**Published:** 2017-12

**Authors:** Daniel A. Smith, Lucy J. Newbury, Guido Drago, Timothy Bowen, James E. Redman

**Affiliations:** a School of Chemistry, Cardiff University, Cardiff CF10 3AT, UK; b Wales Kidney Research Unit, Division of Infection and Immunity, School of Medicine, Cardiff University, Heath Park, Cardiff CF14 4XN, UK; c Cardiff Institute of Tissue Engineering and Repair, Cardiff University, Museum Place, Cardiff CF10 3BG, UK; d Gwent Electronic Materials Ltd, Monmouth House, Mamhilad Pk Est, Pontypool NP4 0HZ, UK

**Keywords:** Femtomolar detection, Urinary microRNAs, Biosensor, Biomarker, Electrochemistry

## Abstract

Altered serum and plasma microRNA (miRNA) expression profiles have been observed in numerous human diseases, with a number of studies describing circulating miRNA biomarkers for cancer diagnosis, prognosis and response to treatment, and recruitment to clinical trials for miRNA-based drug therapy already underway. Electrochemical detection of biomarkers in urine has several significant advantages over circulating biomarker analysis including safety, cost, speed and ease of conversion to the point of care environment. Consequently, much current research is underway to identify urinary miRNA biomarkers for a variety of pathologies including prostate and bladder malignancies, and renal disorders. We describe here a robust method capable of electrochemical detection of human urinary miRNAs at femtomolar concentrations using a complementary DNA-modified glassy carbon electrode. A miR-21-specific DNA hybridisation probe was immobilised onto a glassy carbon electrode modified by sulfonic acid deposition and subsequent chlorination. In our pilot system, the presence of synthetic mature miR-21 oligonucleotides increased resistance at the probe surface to electron transfer from the ferricyanide/ferrocyanide electrolyte. Response was linear for 10 nM–10 fM miR-21, with a limit of detection of 20 fM, and detection discriminated between miR-21, three point-mutated miR-21 sequences, and miR-16. We then demonstrated similar sensitivity and reproducibility of miR-21 detection in urine samples from 5 human control subjects. Our protocol provides a platform for future high-throughput screening of miRNA biomarkers in liquid biopsies.

## Highlights

• A modified glassy carbon electrochemical sensor for microRNAs was developed.
• The electrode allowed detection of femtomolar concentrations of miR-21.
• The method was applied to detection of urinary miR-21.

## 1 Introduction

MicroRNAs (miRNAs) are single-stranded RNA transcripts, typically of 19–23 nucleotides in length. Posttranscriptional regulators of gene expression, miRNAs are currently estimated to target over 60% of the protein coding messenger (mRNAs) encoded by the human genome. Aberrant circulating miRNA expression profiles are associated with cardiovascular disease and numerous types of malignancy, leading to the identification of novel miRNA biomarkers and miRNA-based therapeutic approaches, with recruitment to clinical trials for cancers of the brain, breast and liver underway [1], [2].

In contrast to blood and biopsy collection, urine samples are obtained non-invasively, with minimal inconvenience to the patient. In addition, urine sampling is quicker and safer, leading to reduced test cost and ease of adoption for biochemical laboratory and/or point of care testing. Consequently, numerous studies are underway to identify urinary miRNA biomarkers for renal disorders [3], [4] as well as bladder and prostate cancers [5], [6].

We have recently shown that urinary miRNAs are stabilised by association with i) extracellular vesicles such as exosomes and ii) argonaute 2 protein, and have optimised RT-qPCR-based methods for their isolation and detection [7]. Here, we report the measurement of human urinary miRNA molarity using an optimised electrochemical detection method suitable for development as a point of care test.

Current miRNA detection methods include Northern blotting [8], RT-qPCR [4], [7], microarray [9], surface plasmon resonance [10] and fluorescence-based techniques [11]. These require lengthy protocols together with highly specialised analysts and equipment, limiting their potential use in point of care diagnostics. Detection of nucleic acids and other biomolecules by aptamer-based electrochemistry has been the focus of much recent research interest [12], [13], [14]. Consequently, a number of recent electrochemical miRNA detection studies have demonstrated that high levels of sensitivity can be achieved rapidly and cheaply [15], [16], [17], [18]. However, many of these methods incorporate signal amplification via precious metals and nanoparticles [19], enzymes [20], [21], or four-way junctions [22], or a combination strategy combining fluorescence and electrochemistry [23], and thus remain reliant on specialist knowledge and equipment.

**Scheme 1:**
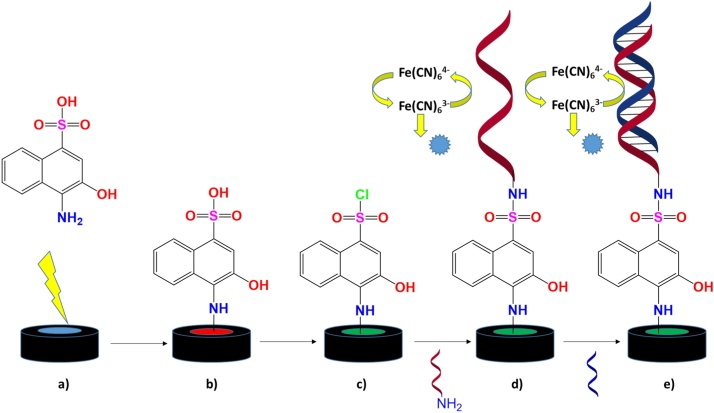
Schematic representation of the fabrication and operation procedure of the biosensor: a, b) A naphthalene sulfonic acid is electrochemically deposited via cyclic voltammetry onto a glassy carbon electrode surface and c) subsequently chlorinated using PCl_5_. d) A DNA oligonucleotide with complementary sequence to the miRNA target (shown in red) is then added and an electrochemical analysis in ferri/ferrocyanide performed. e) Finally, the electrode is hybridised with the target miRNA (blue) and a second comparative electrochemical analysis performed. (For interpretation of the references to colour in this figure legend, the reader is referred to the web version of this article.)

By contrast, following probe fabrication our method requires minimal liquid handling in a straightforward dipstick-style test. Firstly, a glassy carbon electrode is modified with a commercially available naphthalene sulfonic acid derivative to produce a reactive surface, then a DNA probe is attached that will hybridise with complementary target miRNA, as shown in Scheme 1. Electrochemical measurements can then be performed, and the target miRNA concentration obtained. Single-stranded DNA detection has been described elsewhere [24].

We have recently described a robust method to extract and amplify miRNAs from human urine [7]. The use of urine as the sample matrix removes the need for invasive blood sampling procedures used previously [25], [26]. The electrochemical detection procedure we describe herein requires only minimal urine sample treatment and does not require extensive miRNA extraction procedures, toxic and expensive chaotropic reagents, or solvent phase separations, and is therefore more readily applicable to the point of care environment.

## 2 Experimental

### 2.1 Reagents and chemicals

A glassy carbon electrode (3 mm diameter) was obtained from BASi^®^ (Lafayette, USA). All DNA oligonucleotides were synthesised by Sigma-Aldrich Co. Ltd. (Gillingham, UK). Potassium ferri/ferrocyanide, bovine serum albumin (BSA) and Tris HCl were obtained from Sigma-Aldrich. RT-PCR materials were purchased from Qiagen^®^ (Manchester, UK). HPLC purified RNA was purchased from IDT^®^ (Leuven, Belgium), the remaining buffer ingredients and 1-amino-2-naphthol-4-sulfonic acid were purchased from Fisher Scientific^®^ (Loughborough, UK). RNase free water was produced by overnight treatment with 0.1% v/v DEPC and subsequent autoclaving. The DNA and RNA were dissolved in RNase free pH 8.0 TMD buffer consisting of 50 mM Tris-HCl, 20 mM MgCl_2_ and 1 mM dithiothreitol. DNA and RNA samples were stored frozen at −80 °C and denatured at 80 °C for 2 min prior to use, sequence data are provided in the supplementary information (Supplementary table S1).

### 2.2 Instruments

Initial electrochemical measurements to determine sensitivity and optimise sulfonic acid electrodeposition were performed using a PARSTAT-2273 potentiostat/galvanostat and Powersuite^®^ software from Princeton Applied Research^®^. The urine analyses were performed using a PalmSens3^®^ potentiostat supplied by Alvatek^®^ (Tetbury, UK) and data processing performed using PSTrace 4.7 and EIS spectrum analyser.

### 2.3 Electrode preparation and modification

Initially, the electrode was prepared following a modified version of the Wang method [24]. Briefly, a glassy carbon electrode was polished to a mirror-like finish using 3 μm, 1 μm diamond polish and 0.05 μm alumina polish, and then sonicated for 90 s each in acetone, ethanol and water. The electrode was submerged in a solution of 1-amino-2-naphthol-4-sulfonic acid (12 mL, 10 mM) in PBS buffer (25 mM, pH 7.0). This was then prepared as a 3 electrode cell consisting of the glassy carbon electrode as the working electrode, platinum wire as the auxiliary electrode and an Ag/AgCl reference electrode. Cyclic voltammetry was performed between 1.5 V and −0.5 V at a rate of 20 mVs^−1^ to induce electrodeposition until a steady voltammogram was obtained (8 cycles). The electrode was rinsed with distilled water for 30 s, shaken to remove excess water and placed in a 5 mL vial containing PCl_5_ (16.7 mg, 40 mM) in acetone (2 mL) for 30 min [24]. A 10 μL, 1 μM solution of DNA oligonucleotide with a sequence complementary to the target miRNA, dissolved in TMD buffer (50 mM Tris-HCl, 20 mM MgCl_2_, pH 8.0) was heated to a denaturation temperature of 80 °C for 2 min (calculated *T*_m_ 74.7 °C [27]). This solution was then dropped onto the glassy carbon surface and dried under vacuum at 80 °C for 90 min. The electrode was rinsed for 30 s in TMD buffer and electrochemical measurements were taken for use as the initial data points (see 2.4 Electrochemistry). The electrode was then incubated with shaking, for 30 min at 50 °C (Supplementary Fig. S26) in the desired concentration of target miRNA in TMD buffer (1 mL), rinsed with buffer and measurements taken.

### 2.4 Electrochemistry

Analyses for synthetic hsa-miR-21-5p (miR-21) were performed using a PARSTAT-2273 potentiostat and Powersuite^®^ software, urine analyses were performed using a PalmSens3^®^ potentiostat and data processing performed using PSTrace 4.7 and EIS spectrum analyser. Electrochemical measurements were taken before and after probe hybridisation by i) running cyclic voltammograms between 0.6 V and −0.3 V at a scan rate of 100 mV s^−1^ (data not shown) ii) coulometry performed at 0.3 V for 0.1 s, 0.0 V for 2 s and 0.5 V for 2 s and iii) an electrical impedance spectrum (EIS) run at a DC potential of 0.23 V between frequencies of 0.01 Hz to 10 kHz with an AC amplitude of 5 mV. All measurements were taken in 5 mM K_4_[Fe(CN)_6_]/5 mM K_3_[Fe(CN)_6_] in 0.1 M KCl. ΔQ is defined as the change in coulometry signal upon probe hybridisation.

### 2.5 Sodium chloride matrix effects

To investigate sodium chloride matrix effects, the required concentration of miRNA was prepared as before (10^−10^ M, 1 mL) and NaCl was added to a final concentration of 3 mg/mL or 10 mg/mL. The DNA modified electrode was immersed in the miRNA solution at 50 °C for 30 min with shaking and, after rinsing with TMD buffer, the electrochemical measurements were recorded.

### 2.6 Protein matrix effects

BSA (3 mg) was added to miRNA (10^−10^ M) in TMD buffer (1 mL) and the solution shaken to ensure dissolution. Proteinase K (1 mg) and CaCl_2_ (1 mg) were added, and the mixture incubated at 50 °C for 1 h. The solution was incubated with a DNA modified electrode at 50 °C for 30 min with shaking before rinsing in TMD buffer and recording the final electrochemical measurements.

### 2.7 Urine samples

Five urine samples, from anonymised donors, were collected from healthy donors according to guidelines and permission from the Wales Kidney Research Tissue Bank and stored at −80 °C prior to use. Prior to analysis, proteinase K (10 μL, 20 mg/mL) and CaCl_2_ (1 mg) were added to urine (490 μL) and incubated at 50 °C for 10 min. The sample was then passed through a 10 kDa spin filter at 14,000 rcf (12,300 rpm) for 20 min at 20 °C.

### 2.8 Reverse transcription-quantitative PCR (RT-qPCR) analysis

A serial dilution series of miR-21 between 10^−8^ and 10^−14^ M was prepared in TMD buffer. As we have described in detail elsewhere [7], RT-qPCR was then carried out on these dilutions directly, or following extraction using a Qiagen^®^ miRNA extraction kit. Aliquots from the 5 urine samples described above were processed using the Qiagen^®^ miRNA extraction kit. Where appropriate extracts were frozen at −80 °C prior to analysis using standard RT-qPCR protocols (*vide supra*).

### 2.9 Data analysis

All impedance spectra were processed using freely available EIS spectrum analyser [See EIS Spectrum Analyser]. Coulometry and CV data were processed using the software supplied with the respective potentiostat.

## 3 Results and discussion

### 3.1 Initial sensitivity testing of the sensor

**Fig. 1:**
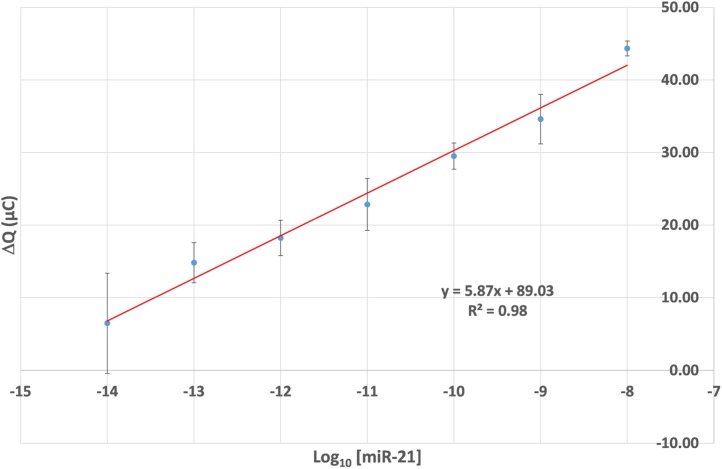
Change in coulometric response between the electrode modified with complementary DNA strand alone and following RNA incubation (ΔQ) with log_10_ miR-21 concentration (M), performed in triplicate. The calculated limit of detection is 2.0 × 10^−14^ M (20 fM) and the regression coefficient (Pearson) is 98%.

This procedure was first performed using solutions of synthetic target miR-21 (Supplementary table S1). The electrode was tested by immersion in a dilution series of synthetic miR-21 solutions spanning 10^−8^ M (10 nM) to 10^−14^ M (10 fM). The turning point from each double step coulometry trace was used for comparison. Changes in coulometric responses (ΔQ) between the electrode modified with a DNA strand alone, and following DNA/RNA hybrid formation, are reported herein due to their linear relationship with log_10_ [miR-21], regression coefficient of 0.984, and low variance in ΔQ when performed in triplicate. The change in signal may be due to formation of RNA/DNA hybrids that inhibit passage of ferro/ferricyanide to the electrode surface by increasing steric occlusion and electrostatic repulsion. These replicates were performed using a freshly prepared electrode surface for each measurement, however electrodes could also be reused up to two times by heat denaturation to remove the RNA with little degradation in the subsequent response (Supplementary Fig. S2). Fig. 1 shows the linear relationship between the charge difference, ΔQ, with log_10_ of the miRNA concentration. By extrapolation to zero signal, the method can potentially detect miR-21 at a concentration of 6 fM. Based upon the standard deviation of measurements of 4 samples containing no RNA, the limit of detection is determined to be 20 fM, which is in the same order of magnitude as the 72 fM quoted by Wang et al. for DNA [24], and close to the 10 fM for RNA suggested by Wu et al. and Tran et al. [28], [29]. The results of electrical impedance spectroscopy, and an overlay of the raw data are shown in the supporting information (Supplementary Figs. S3-5). As a secondary technique, impedance spectroscopy data were robust, with a similar sensitivity to coulometry, albeit with lower R^2^. Differential pulse voltammetry experiments (not shown) were also performed, however these showed an unacceptable level of noise.

### 3.2 Specificity of the sensor

**Fig. 2:**
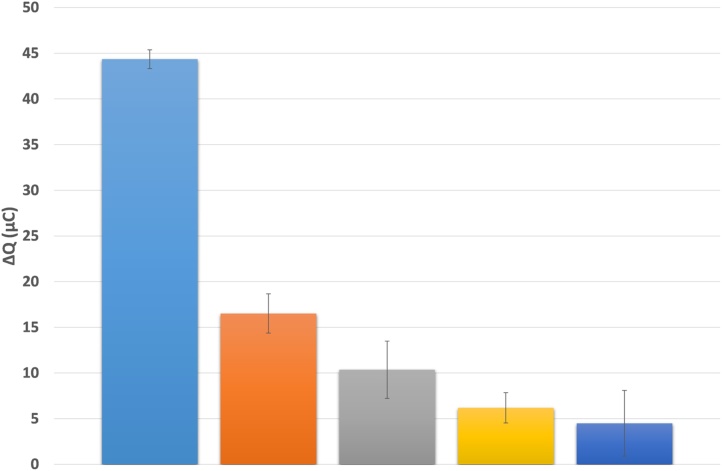
Change in coulometric response (ΔQ) with increasing number of mismatches compared to target miR-21 sequence, performed in triplicate at 10 nM. Shown from left to right, the fully complementary target (blue), 1 mismatch sequence (orange), 2 mismatch sequence (gray), 3 mismatch sequence (yellow), miR-16 (dark blue). (For interpretation of the references to colour in this figure legend, the reader is referred to the web version of this article.)

Since mature miRNAs are very short transcripts, and different miRNAs may vary by only 1 or 2 nucleotides, the selectivity of complementary DNA:target miRNA hybridisation is critical. To test selectivity, we compared the electrochemical signal response using concentrated (10 nM) solutions of synthetic miR-21 with 1, 2 or 3 central and/or peripheral nucleotide changes as well as comparison with the hsa-miR-16-5p (miR-16) sequence (Supplementary table S1). Fig. 2 (Supplementary Fig. S6) shows that the presence of one mismatched nucleotide results in a > 60% reduction in ΔQ, even at this elevated miRNA concentration. Additional mismatches led to further reductions: 2 mismatches by 76%, 3 mismatches by 86% and miR-16 by 90%. These data suggest that our method will be both highly selective and sensitive at the low miRNA concentrations found in human urine [30], [31].

**Fig. 3:**
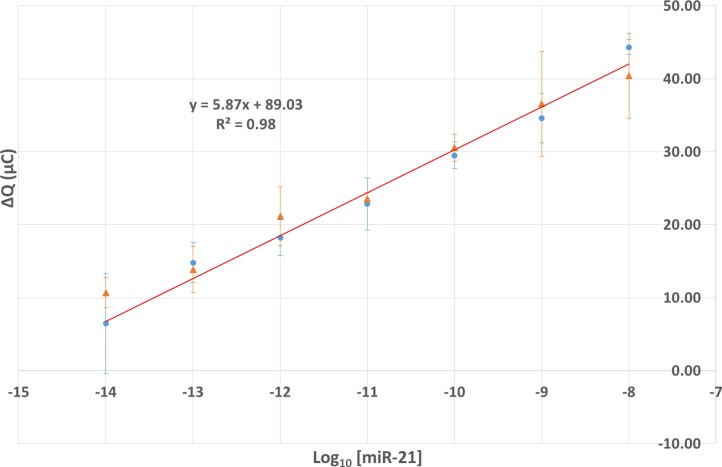
Change in coulometric response (ΔQ) with concentration of miR-21 (blue circles) and miR-16 (orange triangles) with anti-miR-21 and anti-miR-16 DNA probes respectively. Performed in triplicate in a solution of 5 mM K_3_[Fe(CN)_6_]/K_4_[Fe(CN)_6_] in 0.1 M KCl. (For interpretation of the references to colour in this figure legend, the reader is referred to the web version of this article.)

For effective point of care use, our biosensor should be sufficiently flexible to detect and quantify different miRNA targets. We therefore prepared a probe using an oligonucleotide complementary to miR-16, and plots of ΔQ versus log_10_ [miR-16], obtained as before, were overlaid with the corresponding miR-21 results (Fig. 3, Supplementary Fig. S7). Within error the data points are almost coincident, leading to very similar gradients (5.87 ± 0.33 and 5.15 ± 0.23 respectively) in the plots and confirming that the biosensor may readily be manipulated to quantify different miRNA targets. Sensors retained their ability to detect miRNA after treatment at elevated temperatures of up to 50 °C for 24 h (Supplementary Fig. S8).

### 3.3 Urine analogues, human urine and control experiments

We then investigated miR-21 detection using our biosensor in urine mimic aqueous solutions containing salt, urea and protein (bovine serum albumin). While salt and urea show little detrimental effect on the electrochemical response (Supplementary Figs. S9–S11), the presence of protein results in a profound increase in surface impedance (Supplementary Fig. S12). This effect can be attributed to extensive protein adsorption to the electrode surface, therefore interfering with the DNA/RNA response. To digest urinary proteins without full chemical-based miRNA extraction, we incubated urine samples with proteinase K at 50 °C for 10 min, and a response similar to that observed in the absence of protein was seen (Supplementary Fig. S13).

For miRNA analysis in human urine, we wished to compare the electrochemical detection protocols described here with our established RT-qPCR-based methodology. Initial attempts at electrochemical detection of miR-21 in human urine showed an increase in charge transfer resistance at the electrode surface that we attributed to proteinaceous electrode fouling. We have shown previously that exogenous miRNAs added to human urine are degraded rapidly as a result of native RNase activity [7]. We therefore followed the above proteinase K digestion by spin-filtration through a 10 kDa filter to separate miRNAs from remaining high molecular weight biomolecules including RNases, and data obtained after this step indicated an absence of fouling. Control experiments (Supplementary Figs. S14-16) in which miRNA was sequestered by a complementary peptide nucleic acid (PNA), or intentionally cleaved by addition of RNase A, indicated that the responses observed were selective for miR-21 and not due to other components of the urine matrix. An anti-miR-21 probe incubated in urine treated with proteinase K, filtered and then mixed with a PNA complementary for miR-21 gave a negligible response (Fig. S14). When the same solution was tested with an anti-miR-16 probe, a significant change in CV response was obtained (Fig. S15). Finally, an anti-miR-16 probe electrode incubated in a RNase-treated urine solution gave a negligible response (Fig. S16), but following filtration and the addition of synthetic miR-16, the response once again changes significantly. These results provide further evidence that our pre-treatment procedure using proteinase K and filtration effectively prevents protein fouling of the electrode while maintaining its miRNA response.

**Fig. 4:**
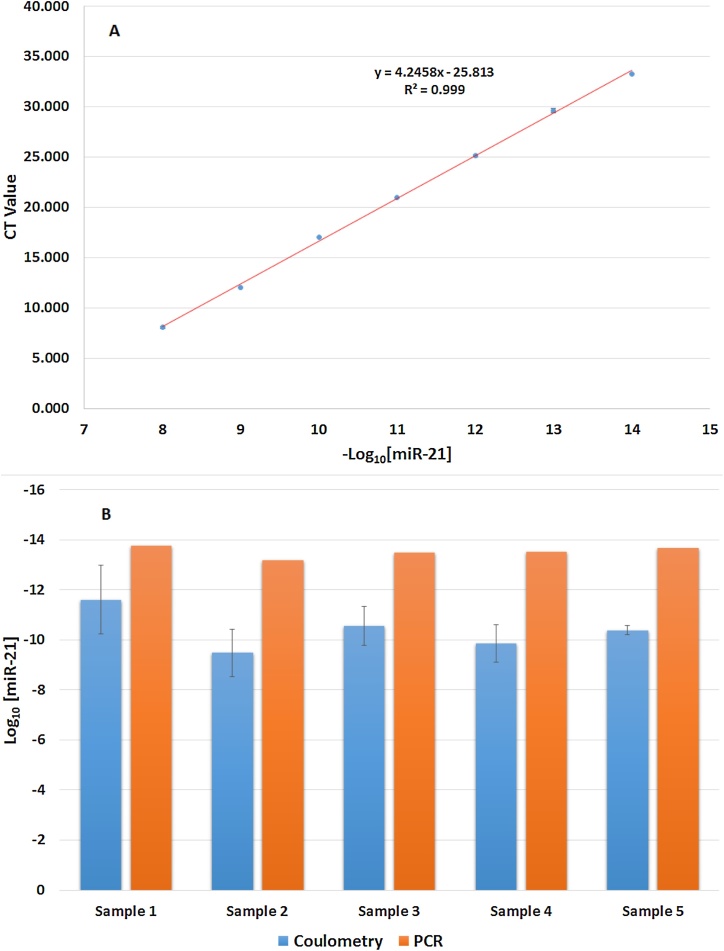
(A) Variation of CT value with miR-21 concentration. (B) Comparison of miR-21 concentration values determined using electrochemical ΔQ (blue, left) and RT-qPCR data (orange, right) in urine samples from 5 different subjects. (For interpretation of the references to colour in this figure legend, the reader is referred to the web version of this article.)

For direct comparison of RNase-free electrochemical- and RT-qPCR-derived miR-21 concentration estimates, we prepared a dilution series of synthetic miR-21 in RT buffer solution (MgCl_2_, Tris HCl) that showed a linear relationship between RT-qPCR threshold cycle and −log_10_ [miR-21]. We then compared electrochemical and RT-qPCR miR-21 detection in 5 human urine samples (Supplementary Figs. S17–S23). RT-qPCR data for urinary miR-21 extracted using an extraction kit were then used to calculate urinary miR-21 concentrations from the calibration curve. MiR-21 was chosen since previous work has shown increased detection of this sequence in urine in chronic kidney disease and acute kidney injury [32], [33]. Fig. 4A and B show the overlaid results of both the RT-qPCR concentration and coulometry analyses.

Despite obtaining overall similarities in concentration ratios for each sample with both techniques, our biosensor detected higher miRNA concentrations than the RT-qPCR analysis. Furthermore, control experiments using known concentrations of synthetic miR-21 produced RT-qPCR data suggesting that the recorded drop in concentration shown in the PCR analysis resulted from losses during sample preparation (Supplementary Figs. S24-25). Since electrochemical detection requires one pipetting step and no chemical extractions, this method may avoid concentration losses and thereby increase sensitivity. The urinary miRNA concentration range we detected (10^−9^–10^−11^ M) falls well within that part of the calibration plot that shows good reproducibility.

Our technique has thus provided a measure of urinary miRNA molarity, and our biosensor has potential for use in future analyses of urinary miRNAs as disease biomarkers.

## 4 Conclusions

In conclusion, we have designed a highly sensitive and specific, straightforward electrochemical biosensor assay for the detection and quantification of urinary miRNAs requiring minimal sample treatment. The electrochemical data obtained from the urinary analysis produced comparable results to RT-qPCR detection, with increased sensitivity, and the biosensor selectively detected two different miRNA sequences. Future investigations using this procedure to quantify urinary miRNAs in healthy and diseased individuals will provide insight into its viability as an applied biosensor. Finally, to expedite commercialisation of our biosensor assay, we are currently testing our method using disposable electrodes that could be supplied pre-modified with DNA to simplify and accelerate the electrochemical analysis in a point-of-care setting.
