# Supplementary material for: Electrochemical detection of urinary microRNAs via sulfonamide-bound antisense hybridisation
Source: Sens Actuators B Chem. 2017 Dec;253:335–41. doi: 10.1016/j.snb.2017.06.069 (PMC5614097; doi:10.1016/j.snb.2017.06.069)
Supplement: Supplementary file 1 [file mmc1.pdf]

**Electrochemical detection of urinary microRNAs via sulfonamide-bound antisense hybridisation**

Daniel A. Smith [a,b,c], Lucy J. Newbury [b,c], Guido Drago [d,†], Timothy Bowen [b,c] and James E. Redman [a,c,*]

[a] School of Chemistry, Cardiff University, Cardiff CF10 3AT, UK

[b] Wales Kidney Research Unit, Division of Infection and Immunity, School of Medicine, Cardiff University, Heath Park, Cardiff CF14 4XN, UK

[c] Cardiff Institute of Tissue Engineering and Repair, Cardiff University, Museum Place, Cardiff CF10 3BG, UK

[d] Gwent Electronic Materials Ltd, Monmouth House/Mamhilad Pk Est, Pontypool NP4 0HZ, UK

* Corresponding author:

James E. Redman, School of Chemistry, Cardiff University, Cardiff CF10 3AT, UK

Tel.: +44-29-2087-6273; fax: +44-29-2087-4030; e-mail: redmanje@cf.ac.uk

[†]Current address:

Perpetuus Carbon Technologies Ltd, Parc Amanwy, New Road, Ammanford SA18 3EZ, UK

E-mails: DAS, smithd16@cf.ac.uk; LJN, newburyl@cf.ac.uk; GD, guidodrago@perpetuuscarbon.com; TB, bowent@cf.ac.uk; JER, redmanje@cf.ac.uk

## Electronic Supplementary Information

## Contents


1. Table S1:- DNA and RNA sequences
2. Figure S2:- Regeneration of the electrode
3. Figure S3:- Electrochemical impedance spectroscopy (EIS)
4. Figure S4-5:- Example overlays of EIS and coulometric data
5. Figure S6:- EIS data for mismatched sequences
6. Figure S7:- EIS data of miR-16 target compared to miR-21
7. Figure S8:- Storage of the probe at increasing temperature for 24 hours
8. Figure S9-S10:- Results of EIS and coulometric analysis with sodium chloride matrix
9. Figure S11:- Result of analysis with urea matrix
10. Figure S12:- Effect of protein and use of proteinase K
11. Figure S13:- Proteinase K treatment of urine
12. Figure S14:- Negative control of urine treatment procedure, using competitive PNA binding
13. Figure S15:- Positive control of urine treatment procedure, using non-complementary PNA
14. Figure S16:- Negative control of urine treatment procedure using RNase A treatment
15. Figure S17:- Resulting concentrations of urine via PCR
16. Figure S18-S23:- Electrochemical measurements of urine
17. Figure S24-S25:- Comparison of direct RT and extraction of known miRNA concentrations
18. Figure S26:- Optimisation of hybridisation time

*Table S1 Sequences of miRNA and complementary DNA strands, with mismatches in bold. The DNA was purchased from Sigma Aldrich® and the RNA from IDT® (Integrated DNA Technologies).*

| Species name | Sequence |
|---|---|
| miR-21 | UAG CUU AUC AGA CUG AUG UUG A |
| miR-16 | UAG CAG CAC GUA AAU AUU GGC G |
| miR-21 1 mm | UAG CUU AUC **G**GA CUG AUG UUG A |
| miR-21 2 mm | UAG CUU AUC **G**GA CUG AUG UUG **C** |
| miR-21 3 mm | **A**AG CUU AUC **G**GA CUG AUG UUG **C** |
| Anti-miR-21 (DNA strand) | 5′NH$_2$-C$_6$-TCA ACA TCA GTC TGA TAA GCT A |
| Anti-miR-16 (DNA strand) | 5′NH$_2$-C$_6$-CGC CAA TAT TTA CGT GCT GCT A |

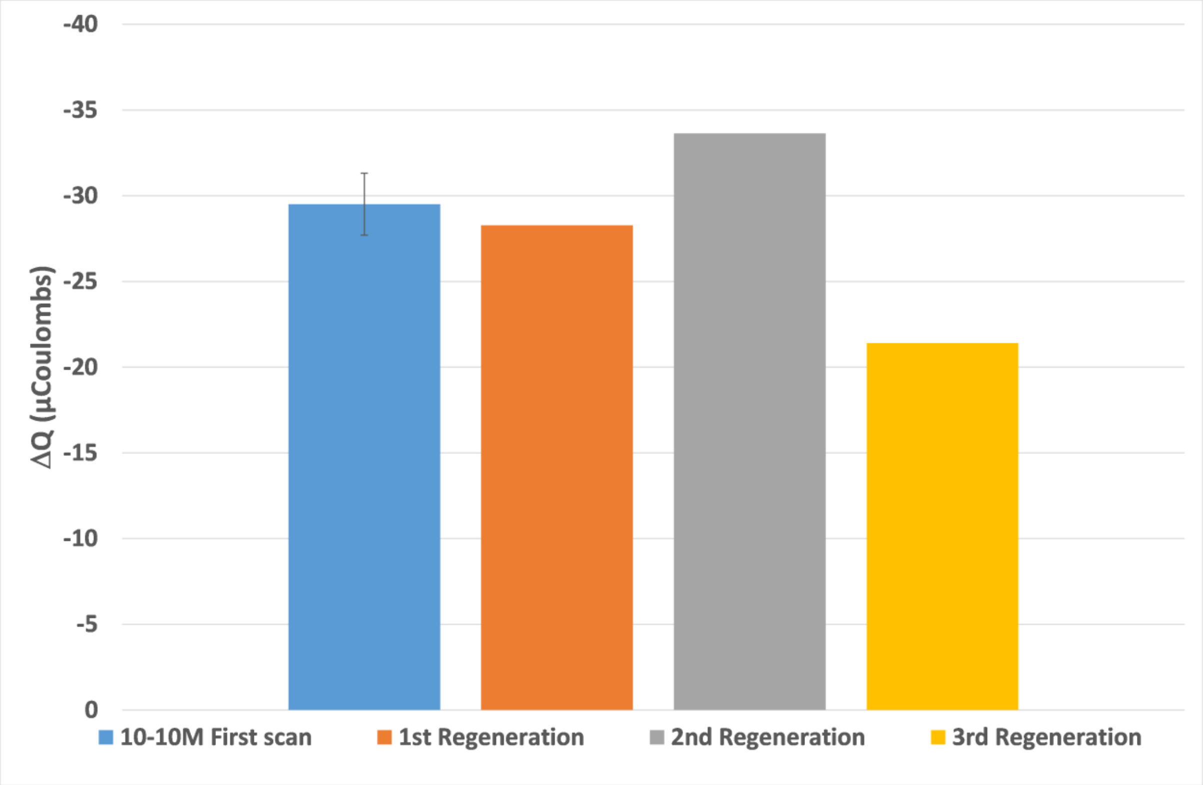


*Figure S2 Change in coulometric response when the electrode is regenerated through a thermal denaturation procedure to remove the hybridised miR-21 followed by another hybridisation event with an identical concentration of $10^{-10}$ M miR-21.*

## Procedure

The electrode was submerged in a 1 mL Eppendorf containing the TMD (50 mM Tris-HCl, 20 mM $MgCl_2$ and 1 mM dithiothreitol pH 8.0) buffer at 95 °C for 20 minutes to remove the RNA from the duplex. Following this, the electrode was submerged into ice cold TMD buffer for 10 minutes. Finally, the electrode was sonicated in the buffer for 2 minutes to remove any residual adsorbed RNA.

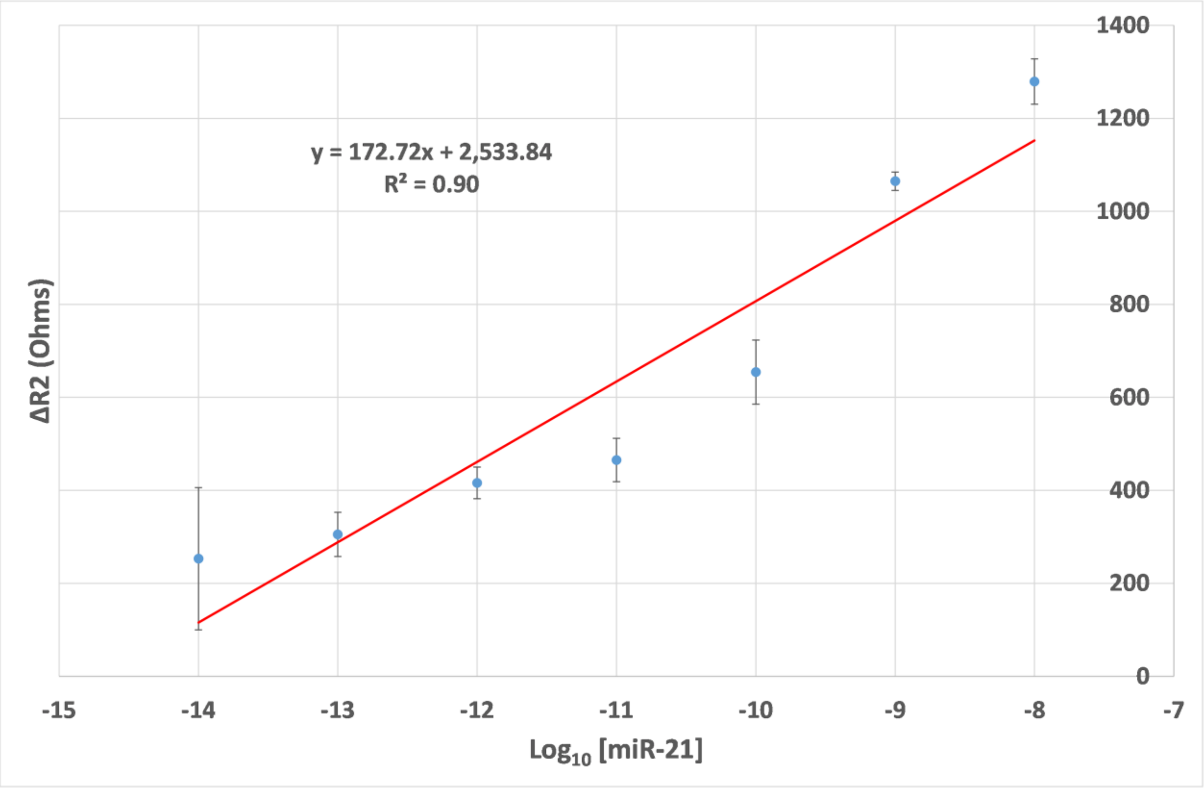


*Figure S3 Change in charge transfer resistance between the complementary strand alone and following RNA incubation (ΔR2) for a glassy carbon electrode modified with anti-miR-21 with variation in concentration of miR-21. Performed in a solution of 5 mM $K_3[Fe(CN)_6]/K_4[Fe(CN)_6]$ in 0.1 M KCl. Performed in triplicate.*

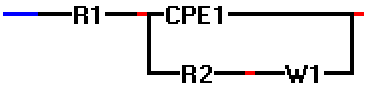


*The equivalent circuit model used to interpret electrical impedance data using EIS spectrum analyser.*

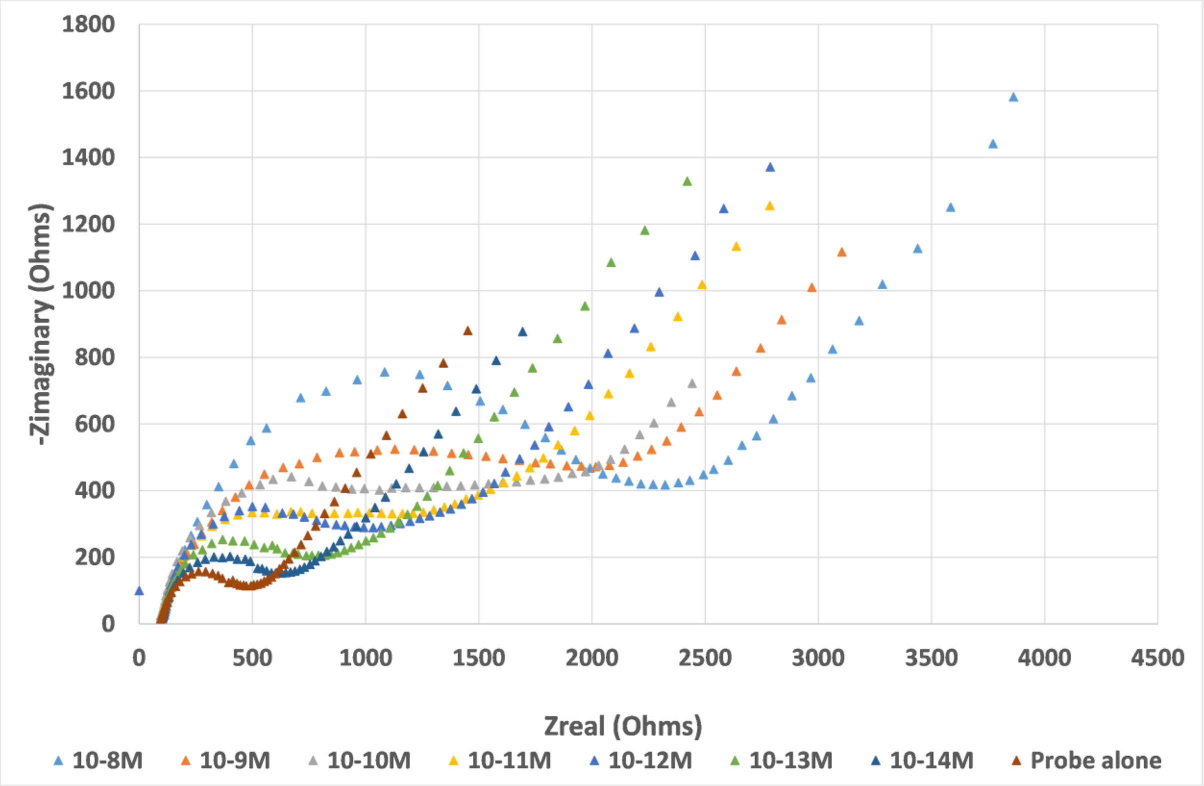


*Figure S4 An overlay of representative Nyquist impedance plots obtained for a glassy carbon electrode modified with anti-miR-21 with variation in concentration of miR-21. Performed in a solution of 5 mM $K_3[Fe(CN)_6]/K_4[Fe(CN)_6]$ in 0.1 M KCl.*

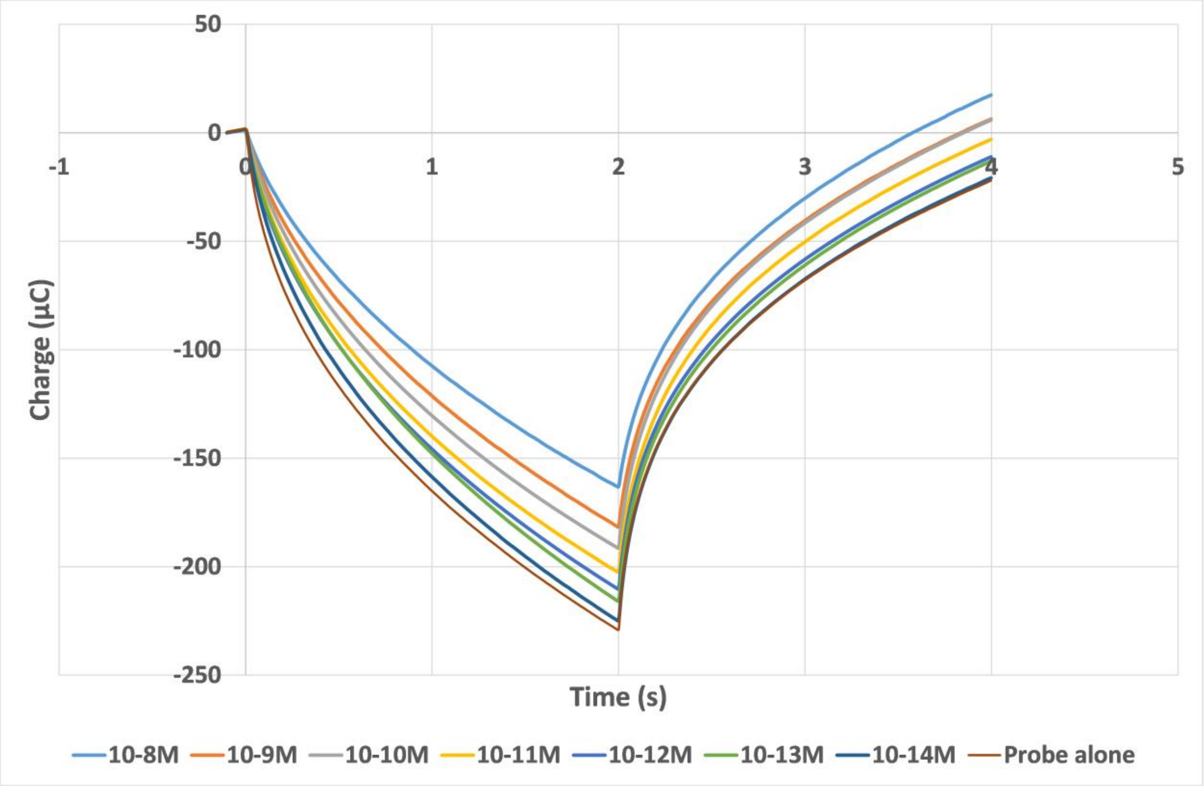


*Figure S5 An overlay of representative coulometry plots obtained for a glassy carbon electrode modified with anti-miR-21 with variation in concentration of miR-21. Performed in a solution of 5 mM $K_3[Fe(CN)_6]/K_4[Fe(CN)_6]$ in 0.1 M KCl.*

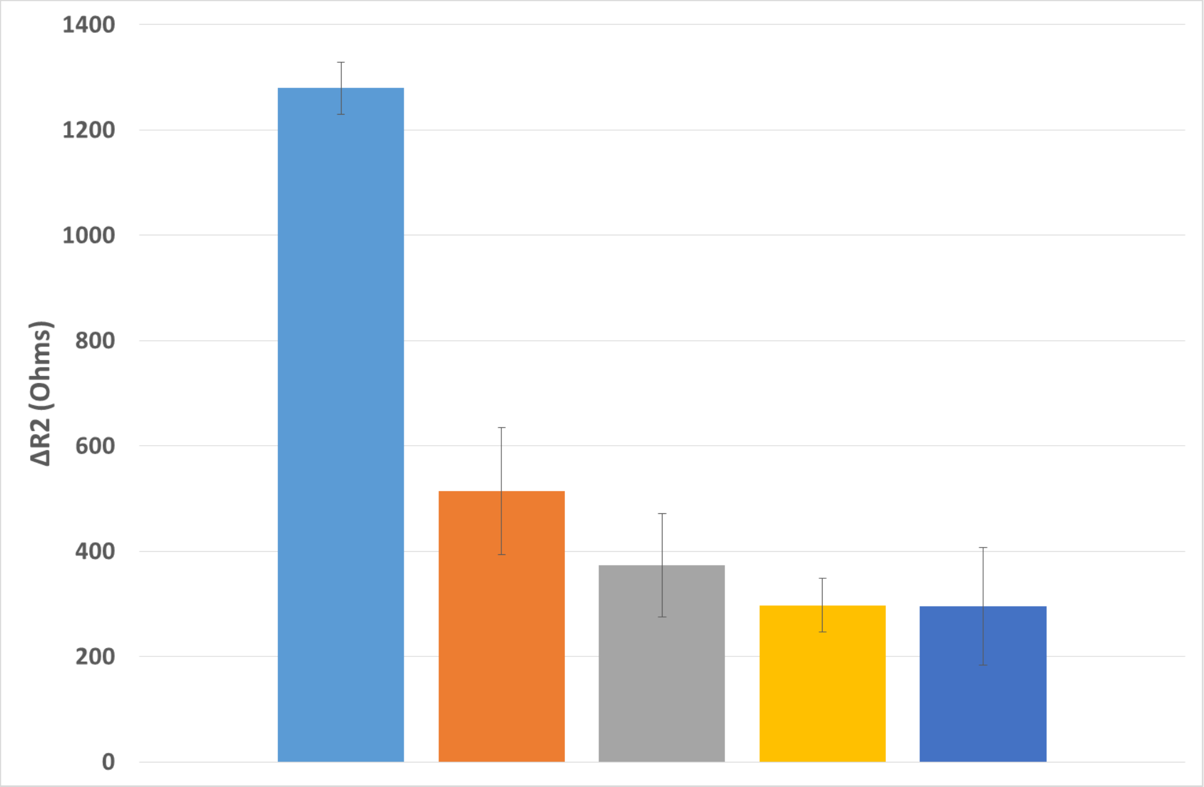


*Figure S6 Change in charge transfer resistance (ΔR2) between the anti-miR-21 DNA probe and DNA/RNA hybrid upon exposure to $10^{-8}$ M solution of mismatched RNA. The $10^{-8}$ M miR-21(light blue), one mismatch (orange), 2 mismatches (gray), 3 mismatches (yellow) and miR-16 (dark blue). Performed in a solution of 5 mM $K_3[Fe(CN)_6]/K_4[Fe(CN)_6]$ in 0.1 M KCl. Performed in triplicate.*

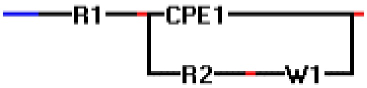

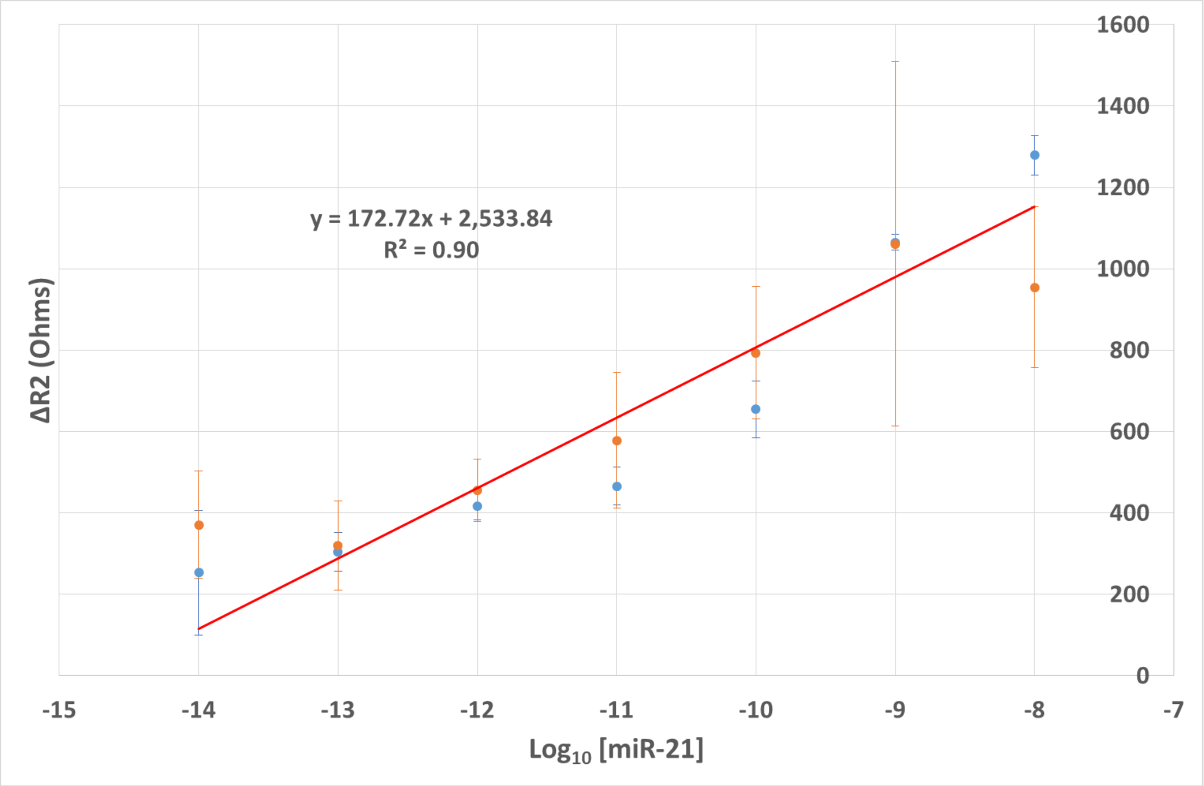


*Figure S7 Change in charge transfer resistance (ΔR2) with concentration of miR-21(blue) and miR-16 (orange) with anti-miR-21 and anti-miR-16 DNA probes respectively. Performed in a solution of 5 mM $K_3[Fe(CN)_6]/K_4[Fe(CN)_6]$ in 0.1 M KCl. Performed in triplicate.*

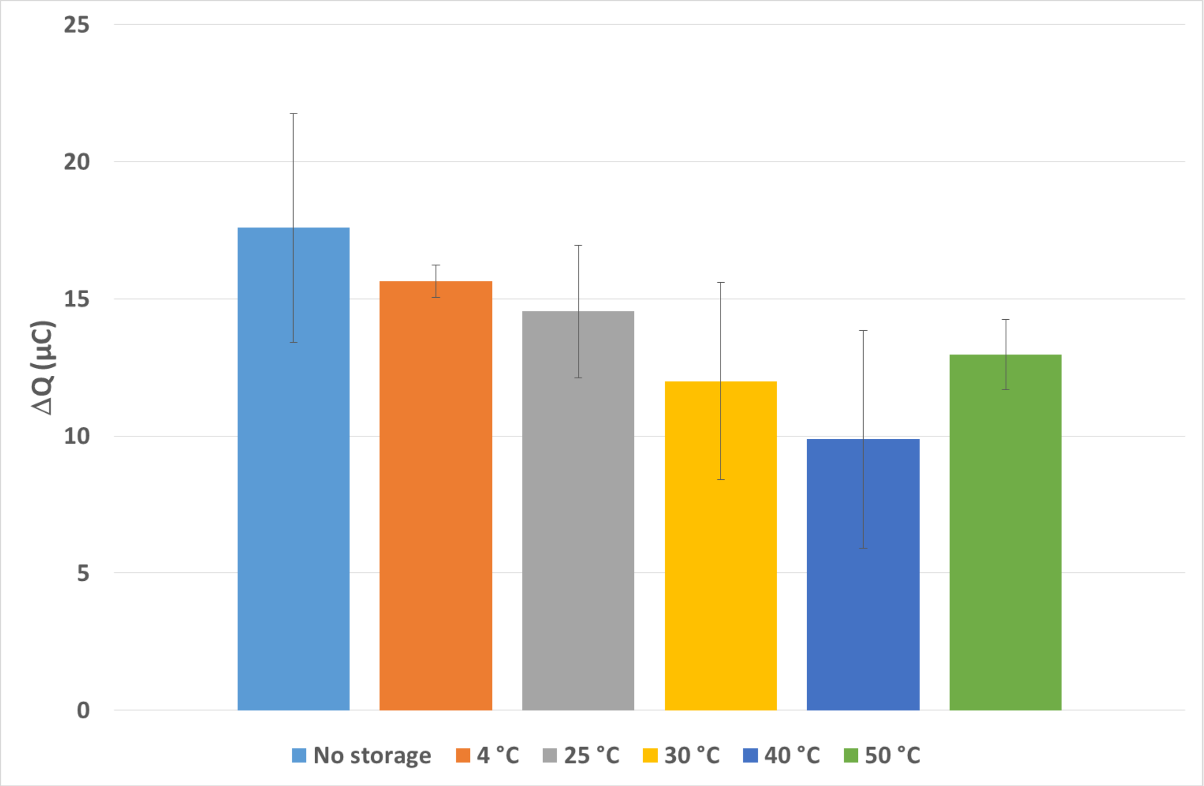


*Figure S8 A bar chart to indicate the change in signal intensity observed for a $10^{-10}$ M solution of miR-21 after storing the DNA modified probe at varying temperatures over 24 hours. Performed in a solution of 5 mM $K_3[Fe(CN)_6]/K_4[Fe(CN)_6]$ in 0.1 M KCl. Performed in triplicate.*

## Note

This data shows that over 50% of the response is maintained after storage of the probe for 24 hours at elevated temperatures of 40 °C and 50 °C.

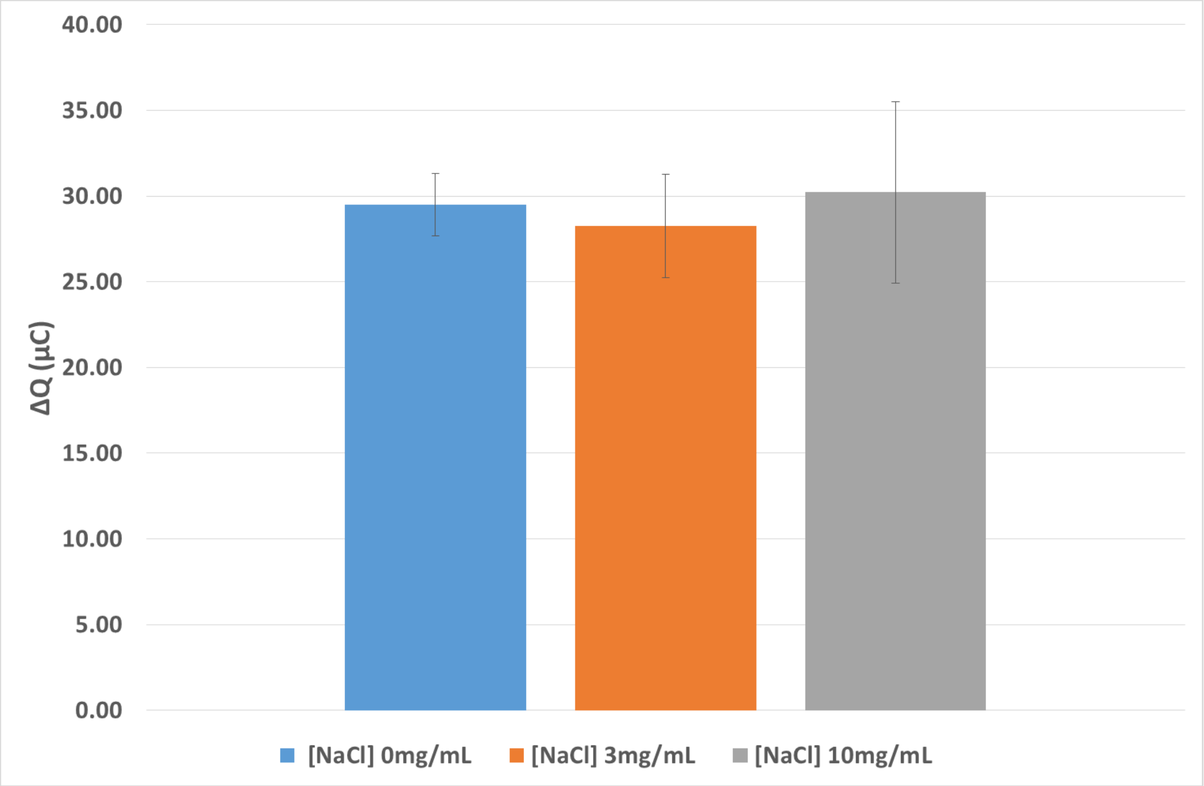


*Figure S9 Coulometric analysis (ΔQ) of miR-21solutions ($10^{-10}$ M) containing sodium chloride using an anti-miR-21 modified electrode. Performed in a solution of 5 mM $K_3[Fe(CN)_6]/K_4[Fe(CN)_6]$ in 0.1 M KCl. Performed in triplicate.*

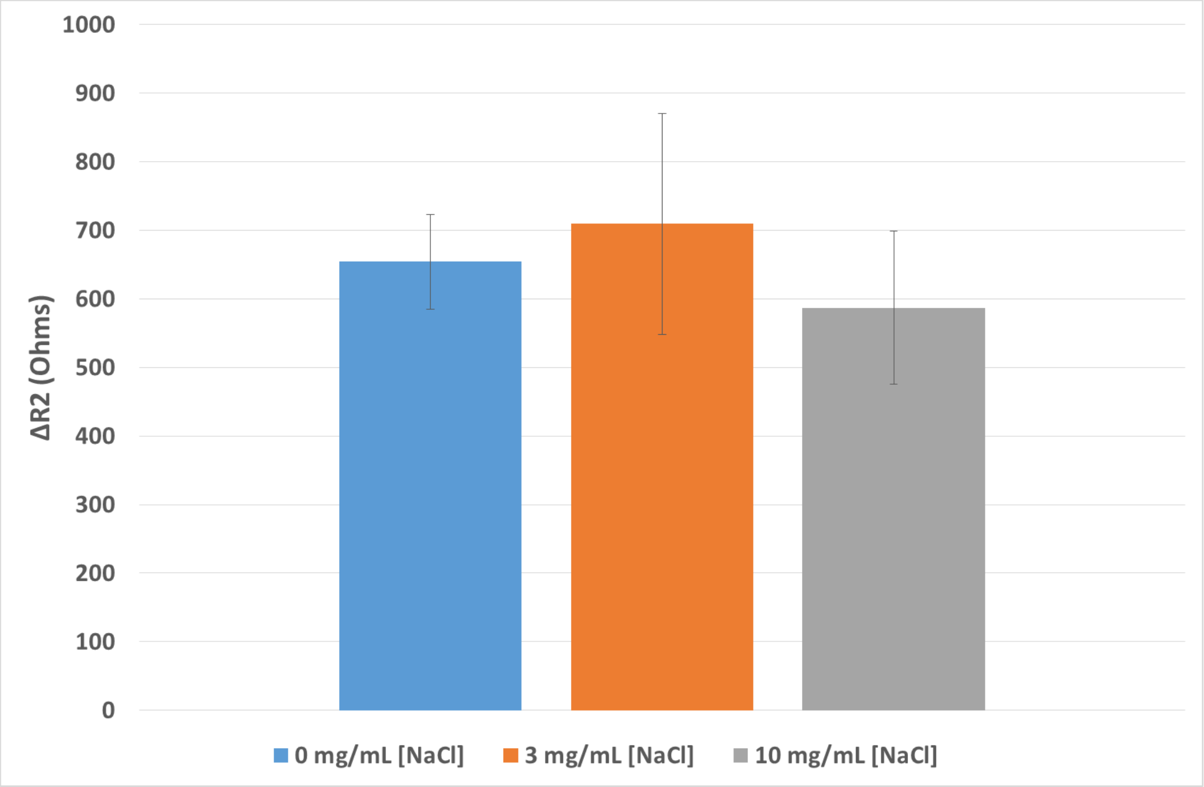


*Figure S10 Charge transfer resistance (ΔR2) of an anti-miR-21 modified electrode exposed to miR-21 solutions ($10^{-10}$ M) containing sodium chloride. Performed in a solution of 5 mM $K_3[Fe(CN)_6]/K_4[Fe(CN)_6]$ in 0.1 M KCl. Performed in triplicate.*

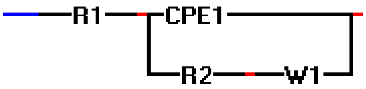

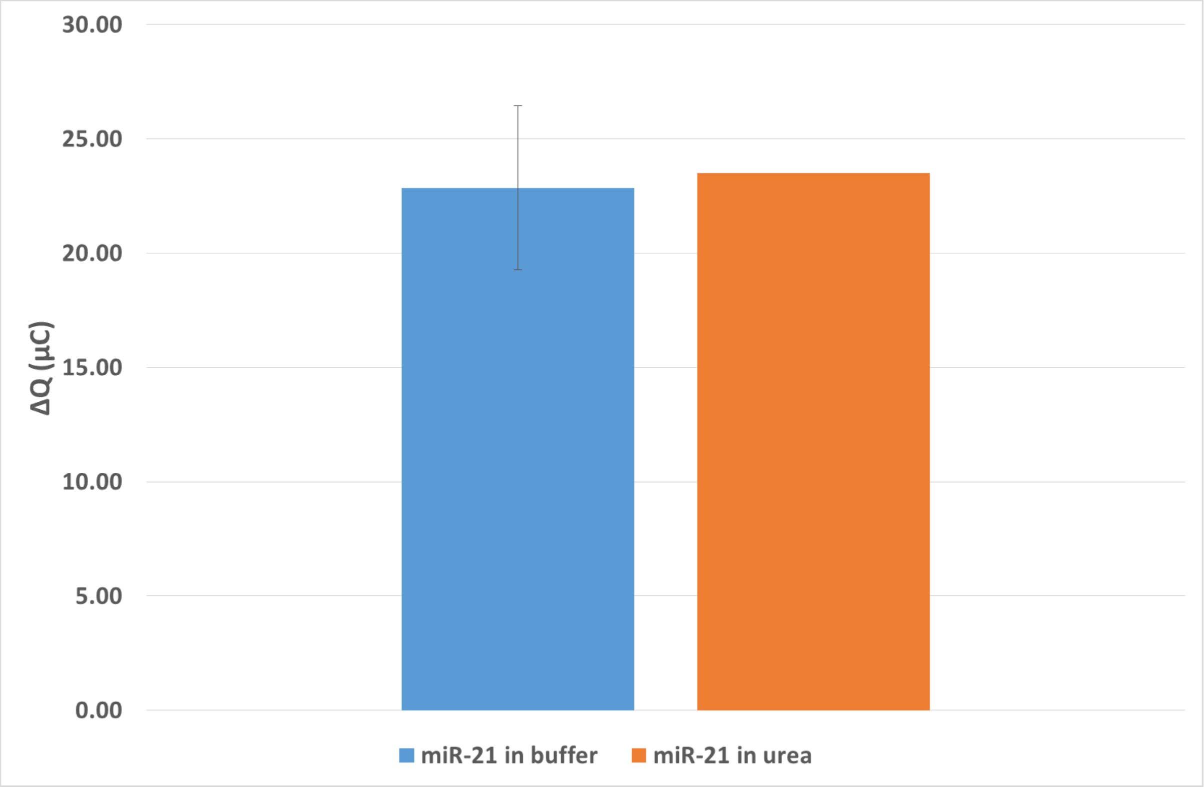


*Figure S11 Coulometric response change (ΔQ) of an anti-miR-21 modified electrode incubated with* $10^{-11}$ *M miR-21 in the presence and absence of urea (155 mM, 9.3 g/L). Performed in a solution of 5 mM* $K_3[Fe(CN)_6]/K_4[Fe(CN)_6]$ *in 0.1 M KCl.*

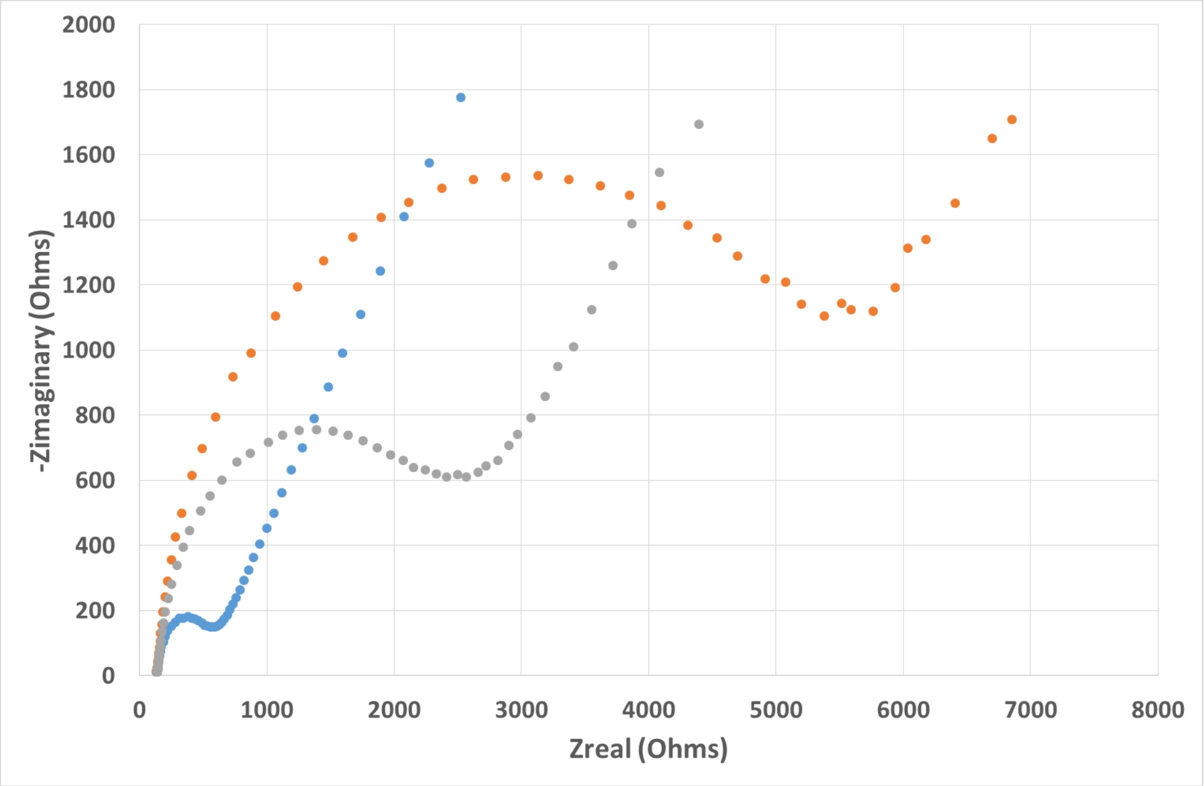


*Figure S12 An overlay of the Nyquist impedance response obtained using an electrode modified with anti-miR-21 (blue), followed by a hybridisation with a miR-21 solution containing 1 mg of BSA (orange) and then following a second incubation in proteinase K for 30 minutes (gray). Performed in a solution of 5 mM $K_3[Fe(CN)_6]/K_4[Fe(CN)_6]$ in 0.1 M KCl.*

**Note**

Protein fouls the electrode surface, however in the presence of proteinase K this effect is greatly reduced.

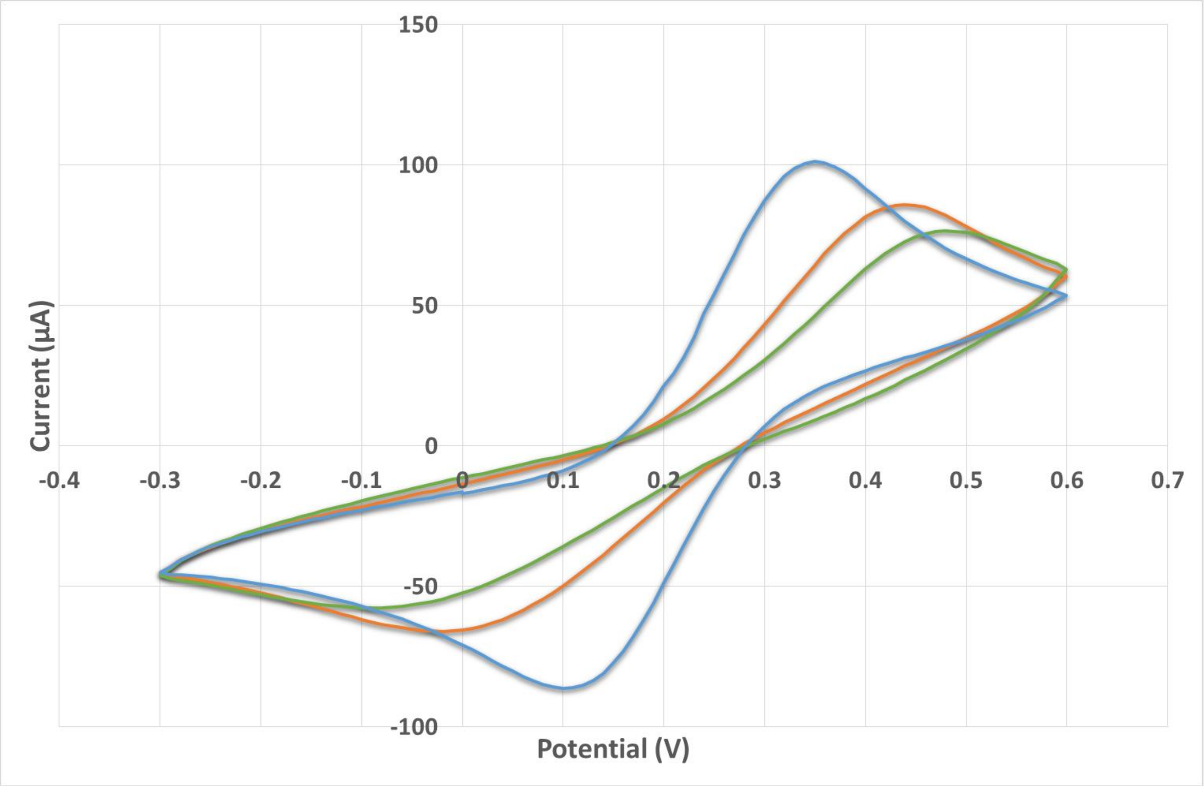


*Figure S13 A CV overlay of the electrode with anti-miR-21 (blue), following incubation with urine (green) and after proteinase K incubation for 10 minutes at 50 °C (orange). Performed in a solution of 5 mM $K_3[Fe(CN)_6]/K_4[Fe(CN)_6]$ in 0.1 M KCl.*

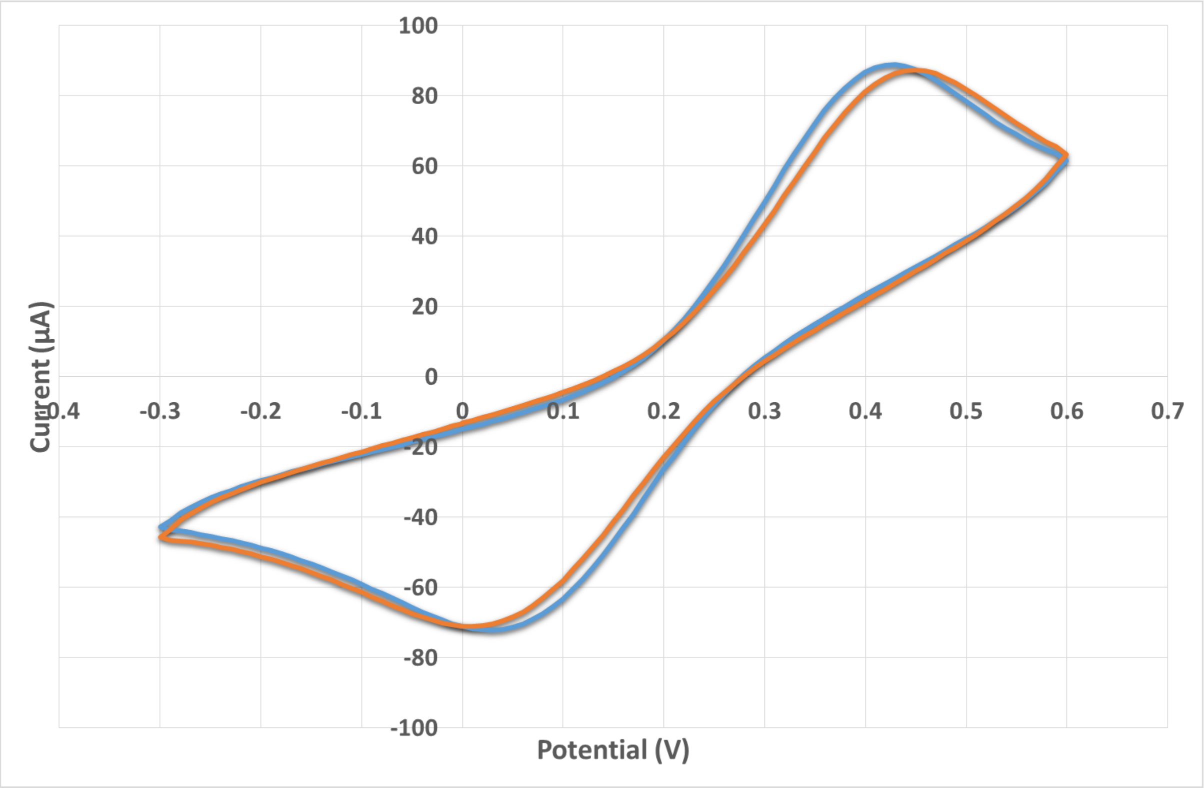


*Figure S14 An overlaid CV of the electrode with anti-miR-21 (orange) and after incubation in filtered urine containing$10^{-9}$ M anti-miR-21 PNA (blue). Performed in a solution of 5 mM $K_3[Fe(CN)_6]/K_4[Fe(CN)_6]$ in 0.1 M KCl.*

**Note**

Treatment of urine with a PNA complementary to the target miRNA resulted in negligible signal. This implies that responses obtained from proteinase K treated and filtered urine are from the miRNA target rather than protein fouling.

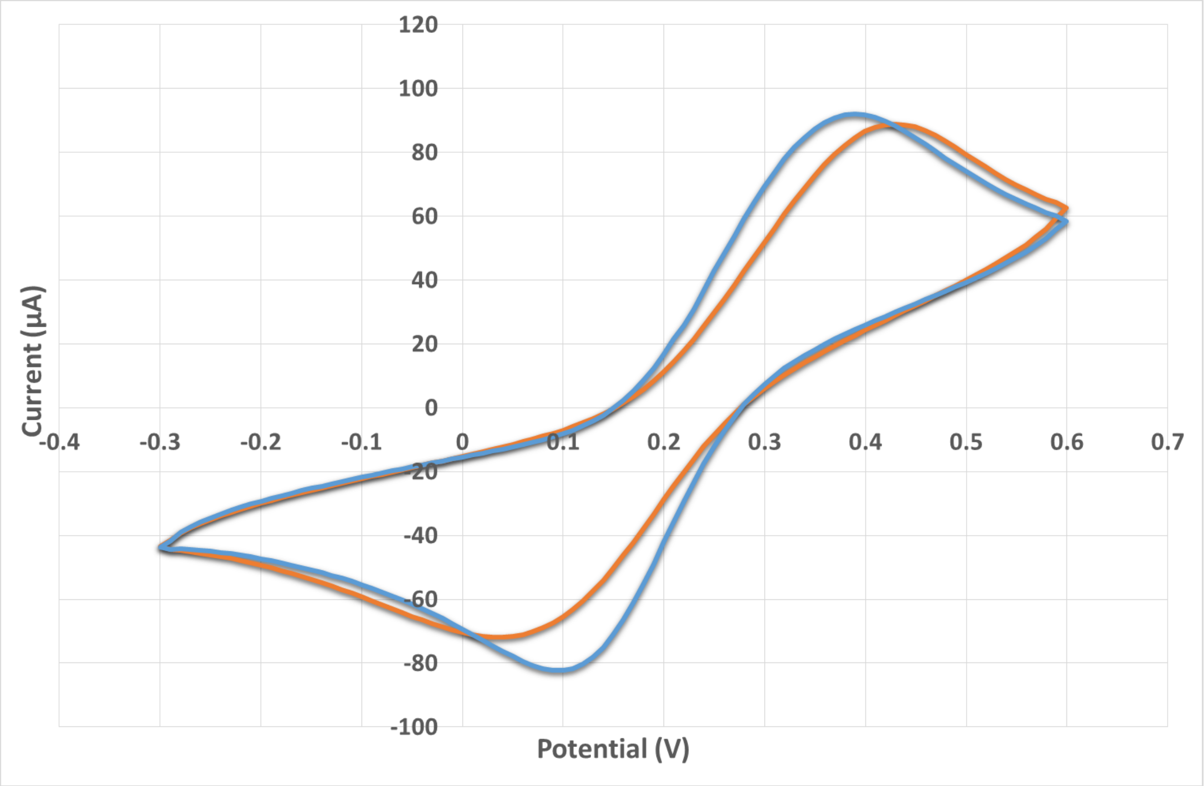


*Figure S15 An overlaid CV of the electrode with anti-miR-16 (blue) and after incubation in filtered urine containing $10^{-9}$ M anti-miR-21 PNA (orange). Performed in a solution of 5 mM $K_3[Fe(CN)_6]/K_4[Fe(CN)_6]$ in 0.1 M KCl.*

**Note**

This experiment shows that the positive control, using a PNA sequence specific to a miRNA that is not the probe target, resulted in a significant signal change being obtained upon hybridisation. A further implication is that the responses obtained from the proteinase K treated and filtered urine are from the specific miRNA target.

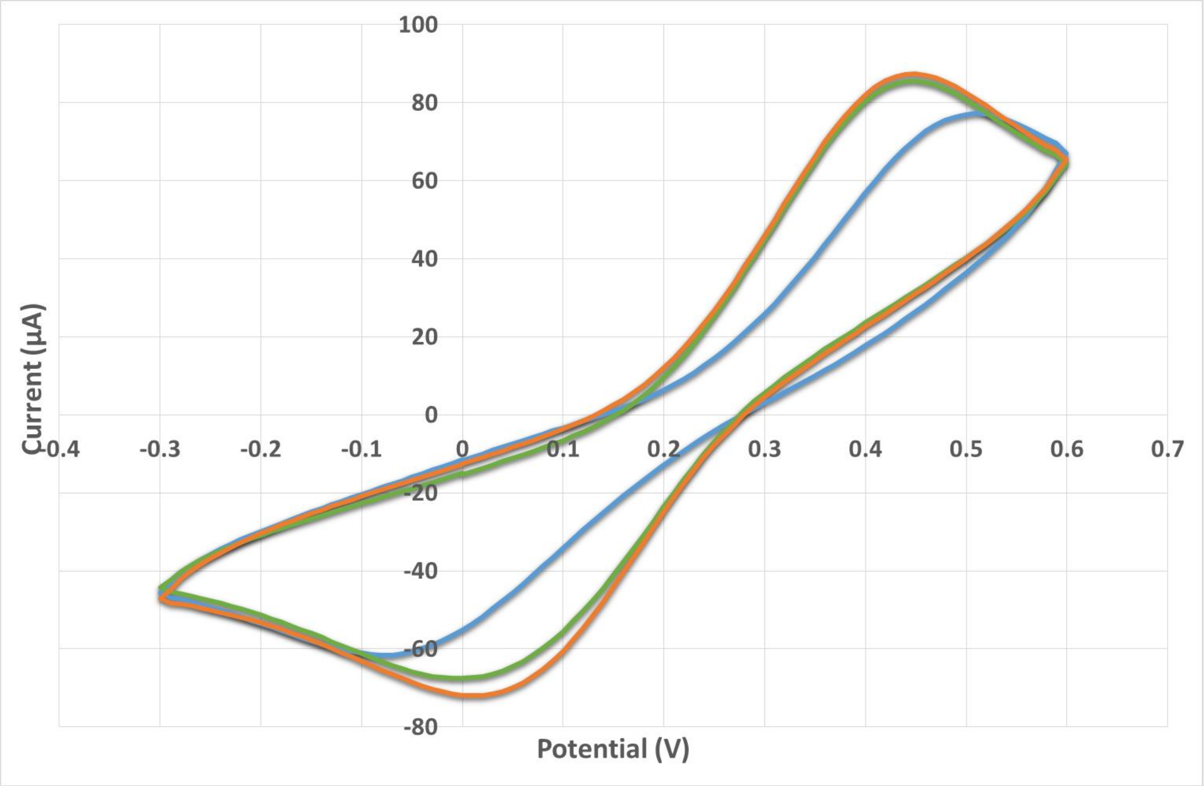


*Figure S16 An overlaid CV of the electrode with anti-miR-16 (orange), after incubation with RNase A treated urine for 20 minutes prior to proteinase K digestion and filtration (green) and following a 'spike' of the filtered urine solution with $10^{-11}$ M miR-16 (blue). Performed in a solution of 5 mM $K_3[Fe(CN)_6]/K_4[Fe(CN)_6]$ in 0.1 M KCl.*

**Note**

Addition of RNase degrades RNA in the urine resulting in negligible current change. Finally, a miR-16 'spike' is used to show that the signal can be restored, and also that the RNase used previously (and likely any originally present in the urine) is removed upon filtration through the 10 kDa spin filter.

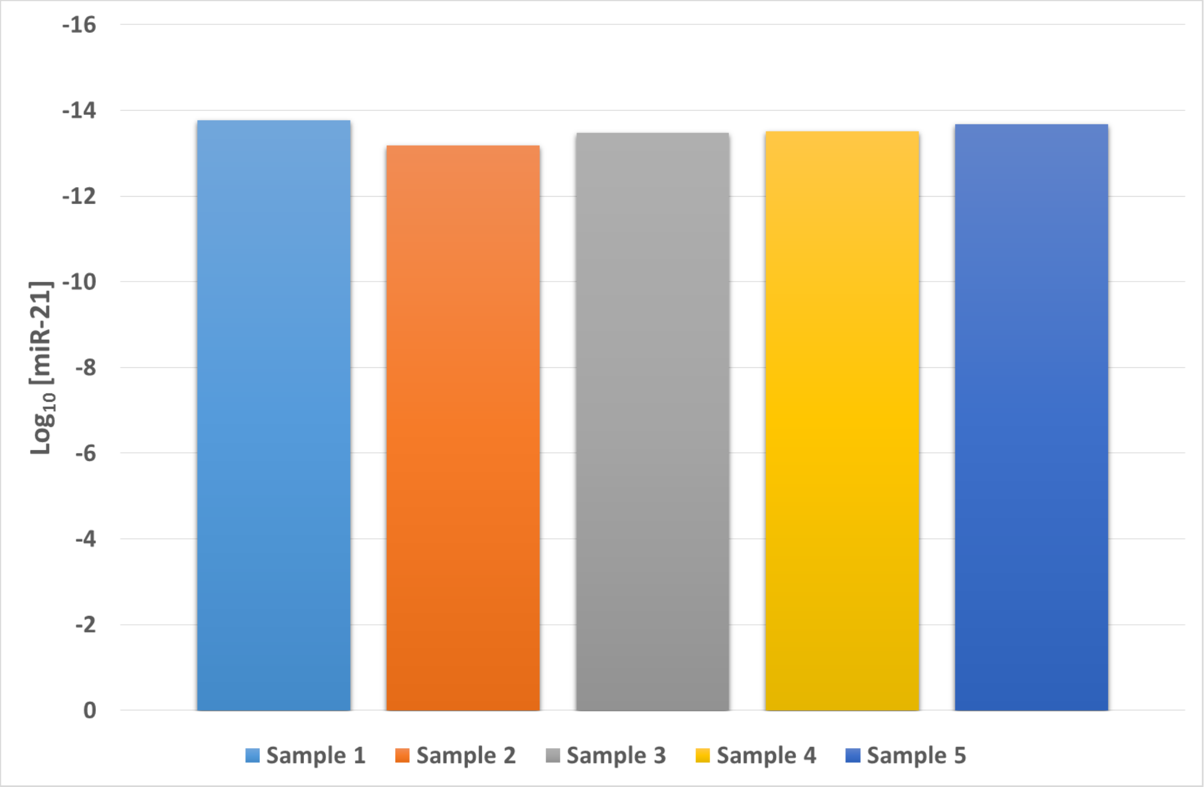


*Figure S17 The results of urine sample concentrations obtained by comparing the CT values of each urine sample to the calibration plot from the samples of known concentration. Performed using miR-21 specific primers.*

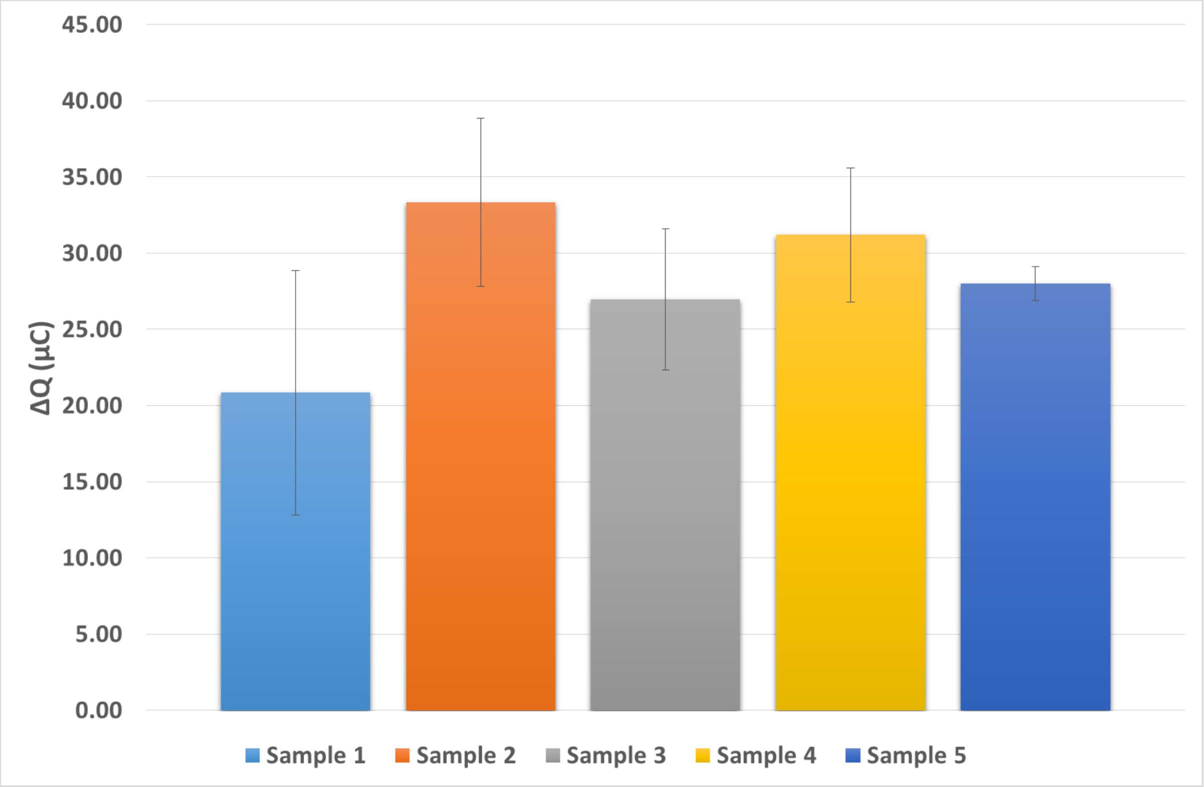


*Figure S18 Change in coulometric response (ΔQ) obtained using an electrode modified with anti-miR-21 for 5 urine samples. Performed in a solution of 5 mM $K_3[Fe(CN)_6]/K_4[Fe(CN)_6]$ in 0.1 M KCl. Performed in triplicate.*

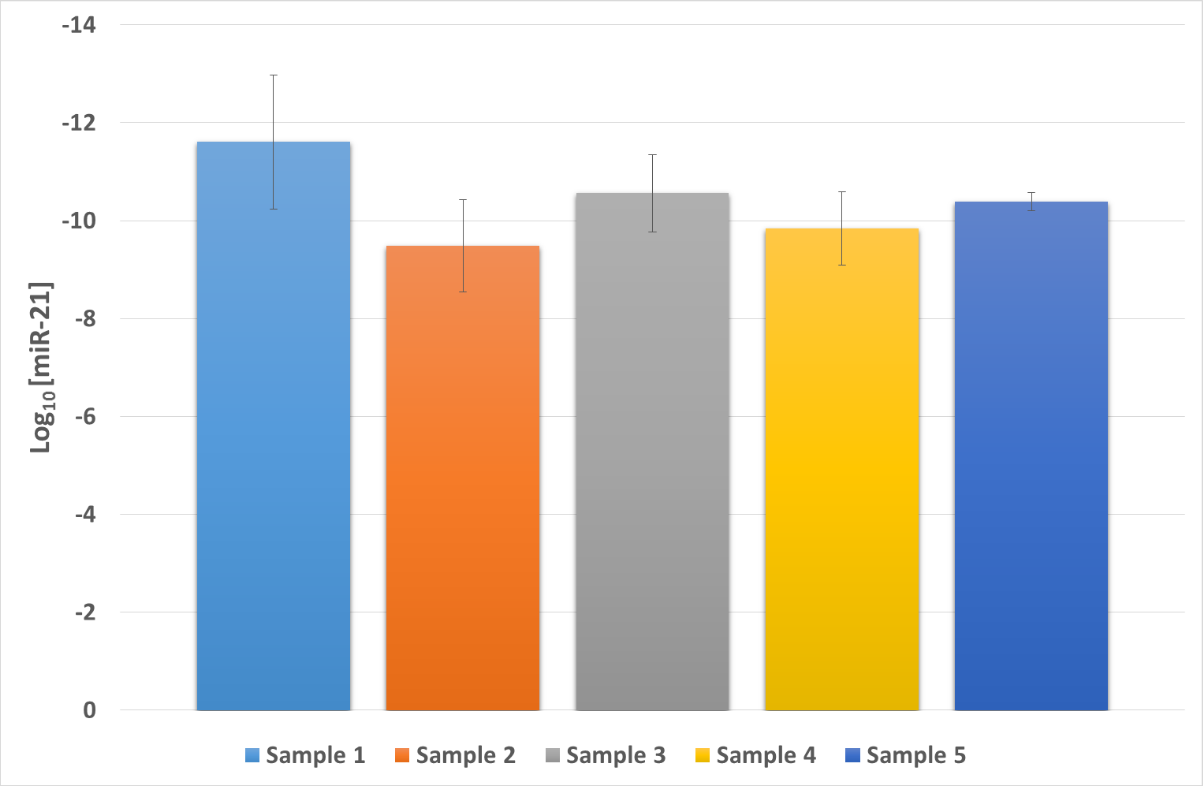


*Figure S19 Coulometric responses of Figure S16 converted into concentration using the calibration plot shown in figure 1 of the manuscript.*

**Note**

Higher bar is a lower concentration.

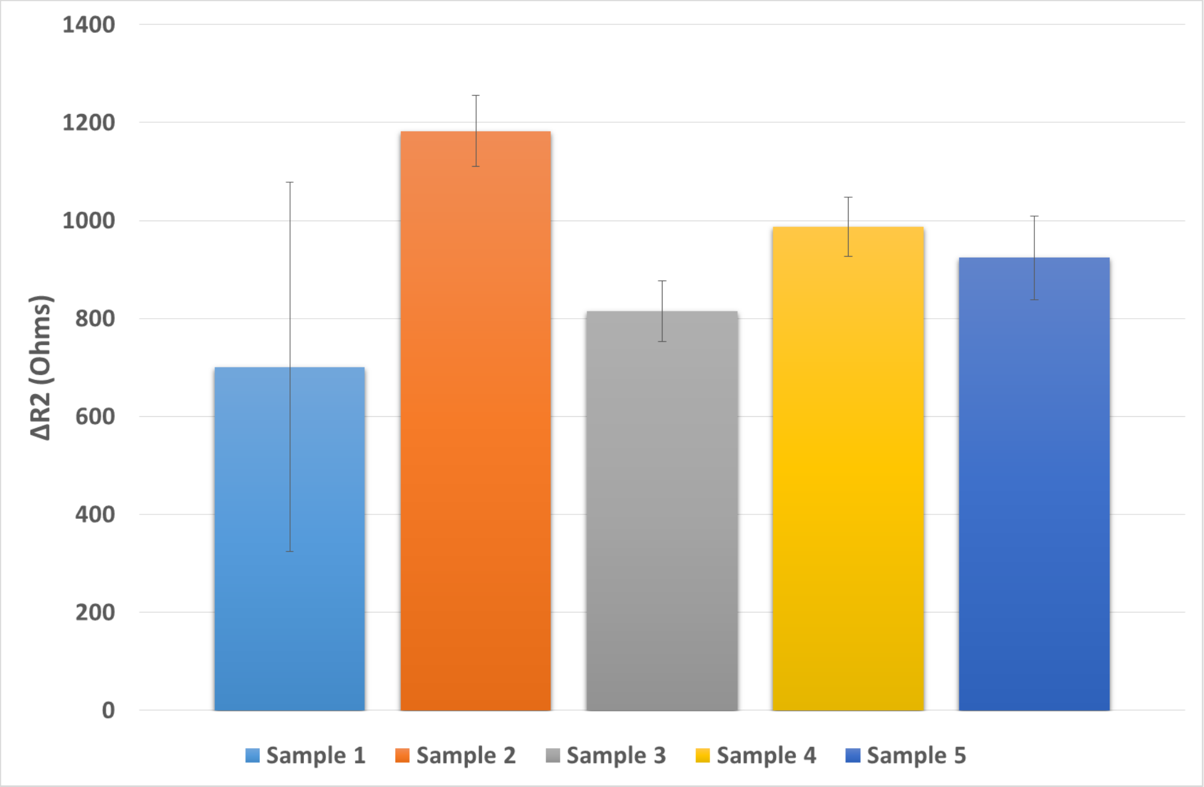


*Figure S20 Change in charge transfer resistance (ΔR2), obtained using an electrode modified with anti-miR-21 for 5 urine samples. Performed in a solution of 5 mM $K_3[Fe(CN)_6]/K_4[Fe(CN)_6]$ in 0.1 M KCl. Performed in triplicate.*

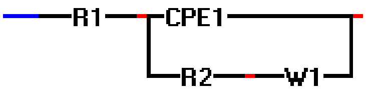

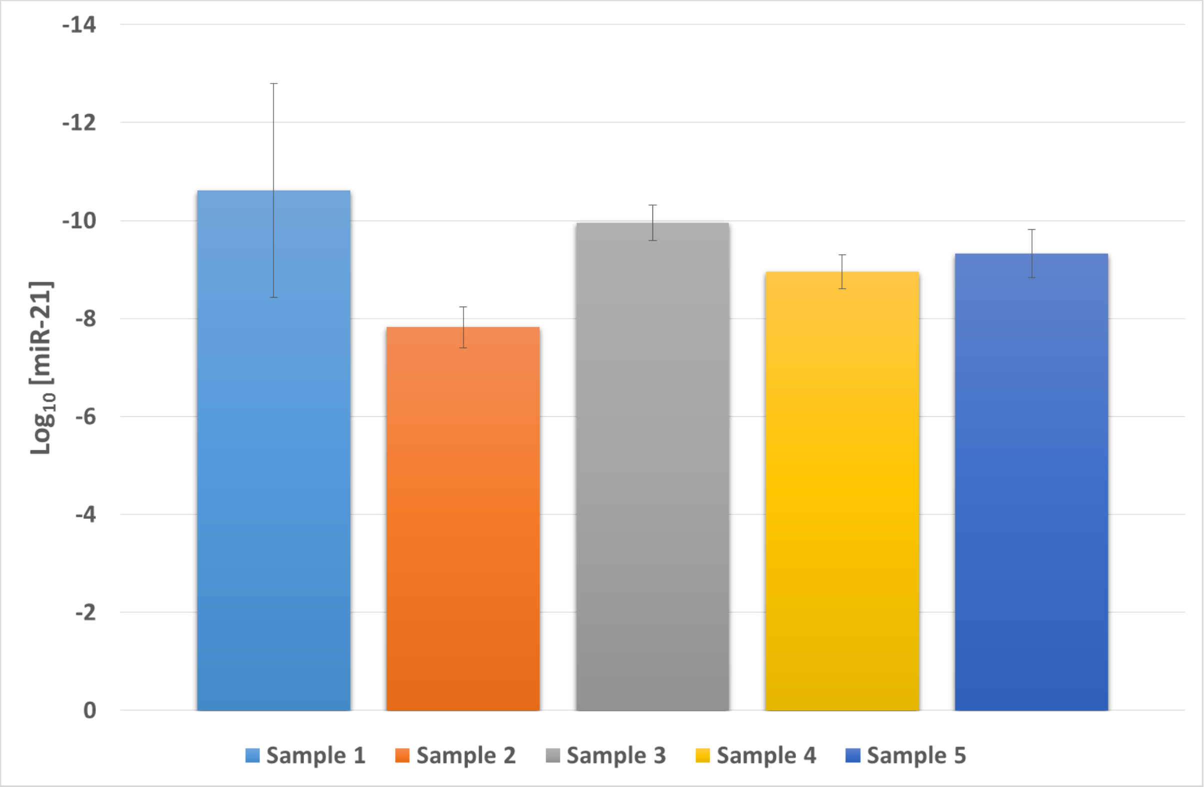


*Figure S21 Charge transfer resistance (ΔR2) of Figure S18 converted into concentration using the calibration plot shown in figure S2.*

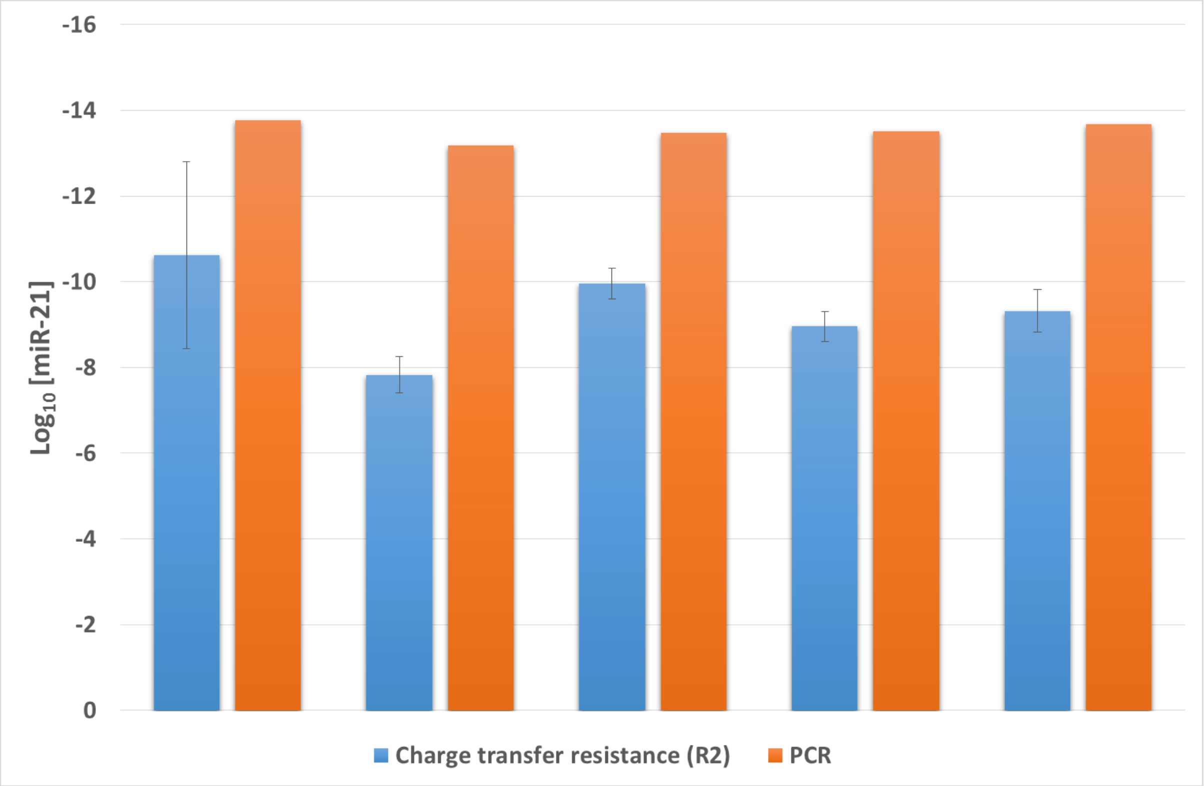


*Figure S22 Charge transfer resistance (ΔR2) plot converted into concentration (blue) using the calibration plot shown in figure S2 overlaid with the PCR concentration data (orange) for the same samples.*

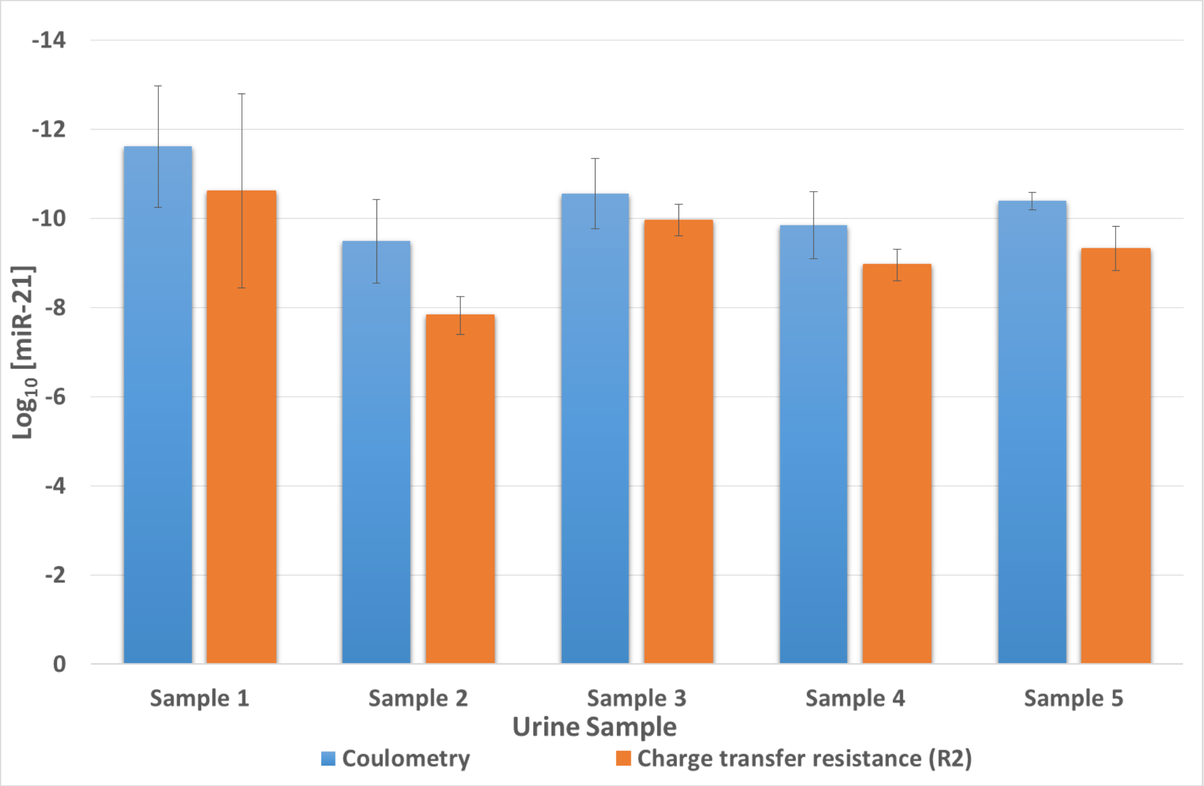


*Figure S23 An overlay of the result of all electrochemical techniques used to analyse the urine samples.*

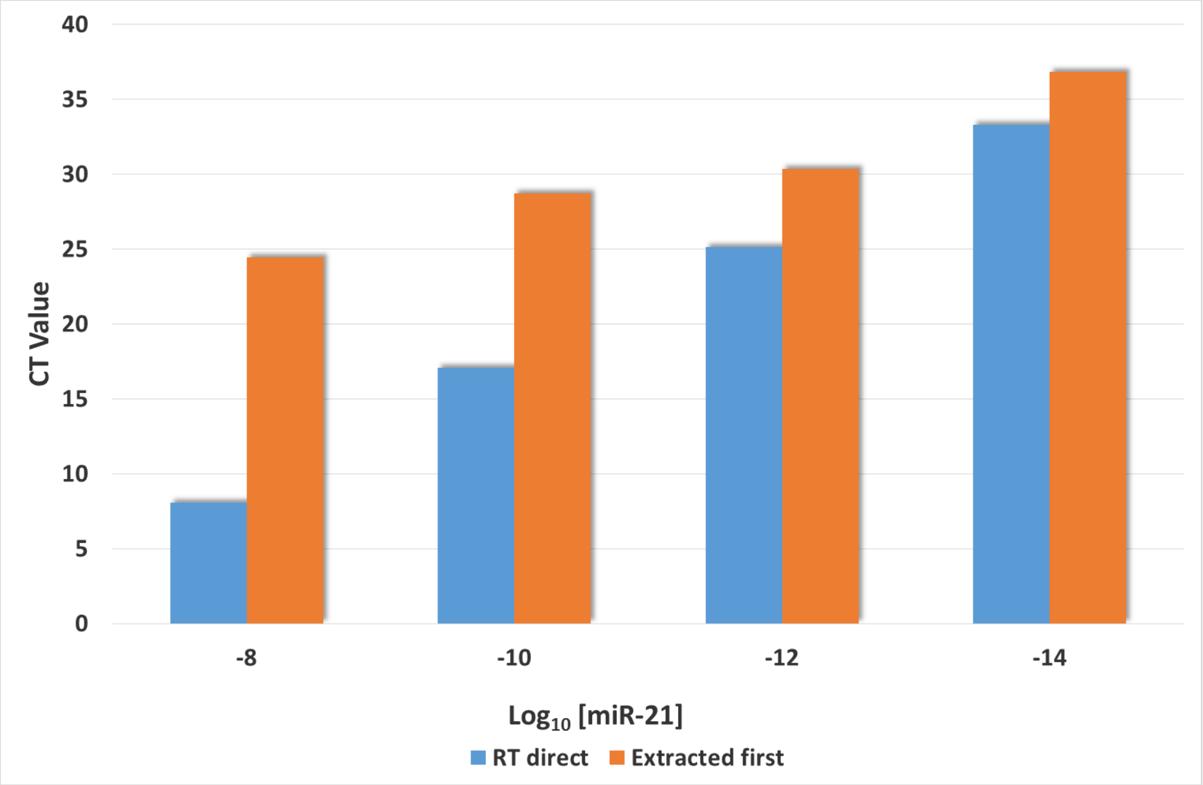


*Figure S24 The difference in CT values obtained upon PCR amplification of a range of synthetic miR-21 solutions, one set where RT was directly performed, and one where an extraction step was performed first.*

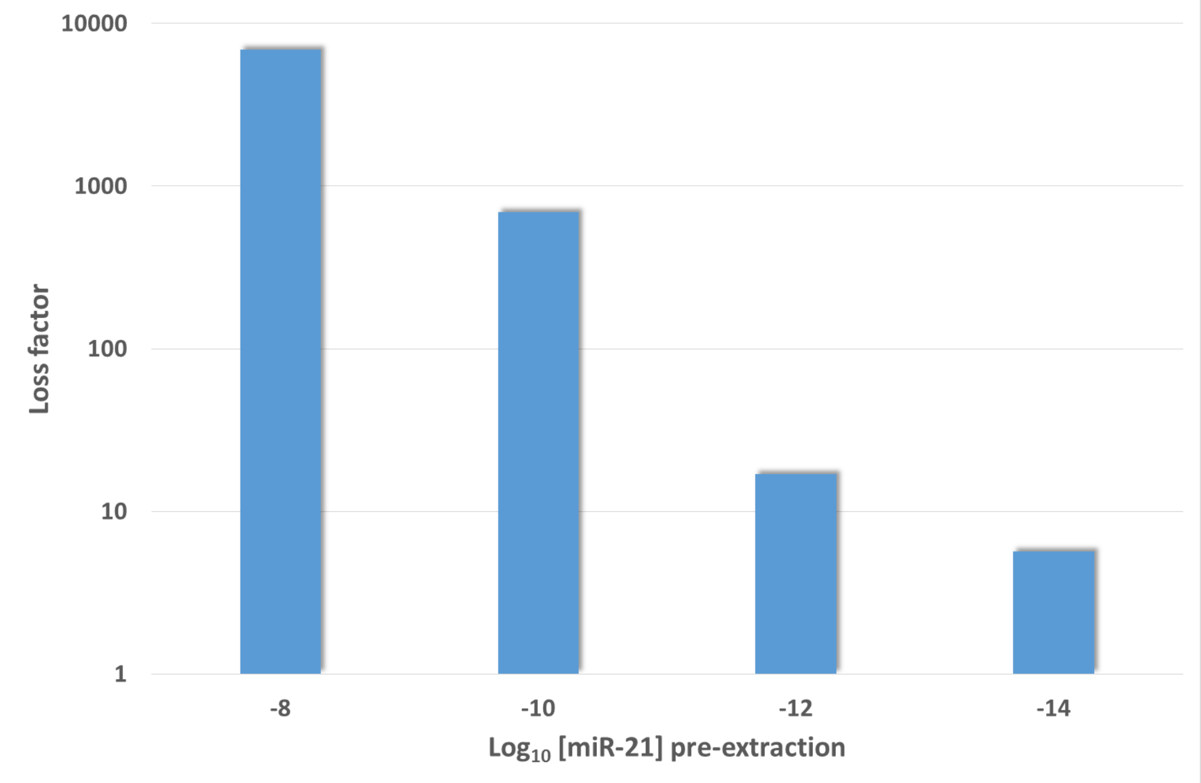


*Figure S25 The loss of concentration observed when an extraction step is performed prior to RT-qPCR amplification of a synthetic miR-21 solution compared to one where the RT is performed using the solution directly.*

**Note**

The difference in RT-qPCR response, between extracted and directly analysed samples, decreased with decreasing initial miRNA concentration. With an initial concentration of $10^{-8}$ M, a decrease of approx. 7000× upon extraction is observed; this is lowered to approx. 700 at $10^{-10}$ M and further decreased at $10^{-12}$ and $10^{-14}$ M. The electrochemical analyses do not require extraction and so are not susceptible to these losses at higher miRNA concentrations.

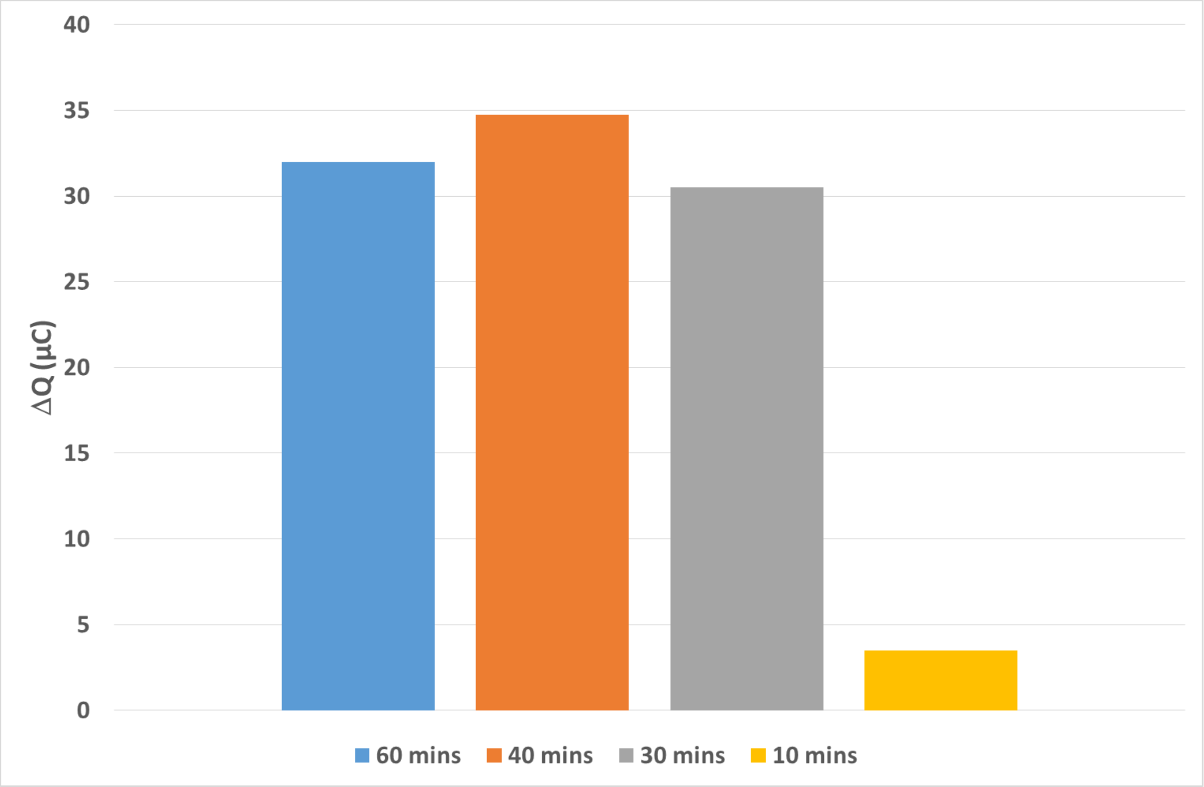


*Figure S26 Coulometric response change (ΔQ) as a function of incubation time for $10^{-10}$ M miR-16. The optimised time is 30 minutes.*
